# Electron transfer pathways in a light, oxygen, voltage (LOV) protein devoid of the photoactive cysteine

**DOI:** 10.1038/s41598-017-13420-1

**Published:** 2017-10-17

**Authors:** Benita Kopka, Kathrin Magerl, Anton Savitsky, Mehdi D. Davari, Katrin Röllen, Marco Bocola, Bernhard Dick, Ulrich Schwaneberg, Karl-Erich Jaeger, Ulrich Krauss

**Affiliations:** 1 Institut für Molekulare Enzymtechnologie, Heinrich-Heine-Universität Düsseldorf, Forschungszentrum Jülich, 52426, Jülich, Germany; 2 Institut für Physikalische und Theoretische Chemie, Universität Regensburg, 93053, Regensburg, Germany; 3 Max Planck Institute for Chemical Energy Conversion, 45470, Mülheim an der Ruhr, Germany; 4 Lehrstuhl für Biotechnologie, RWTH Aachen University, Worringerweg 3, 52074, Aachen, Germany; 5 DWI-Leibniz Institute for Interactive Materials, Forckenbeckstraße 50, 52056, Aachen, Germany; 6 IBG-1: Biotechnologie, Forschungszentrum Jülich, 52426, Jülich, Germany

## Abstract

Blue-light absorption by the flavin chromophore in light, oxygen, voltage (LOV) photoreceptors triggers photochemical reactions that lead to the formation of a flavin-cysteine adduct. While it has long been assumed that adduct formation is essential for signaling, it was recently shown that LOV photoreceptor variants devoid of the photoactive cysteine can elicit a functional response and that flavin photoreduction to the neutral semiquinone radical is sufficient for signal transduction. Currently, the mechanistic basis of the underlying electron- (eT) and proton-transfer (pT) reactions is not well understood. We here reengineered pT into the naturally not photoreducible iLOV protein, a fluorescent reporter protein derived from the *Arabidopsis thaliana* phototropin-2 LOV2 domain. A single amino-acid substitution (Q489D) enabled efficient photoreduction, suggesting that an eT pathway is naturally present in the protein. By using a combination of site-directed mutagenesis, steady-state UV/Vis, transient absorption and electron paramagnetic resonance spectroscopy, we investigate the underlying eT and pT reactions. Our study provides strong evidence that several Tyr and Trp residues, highly conserved in all LOV proteins, constitute the eT pathway for flavin photoreduction, suggesting that the propensity for photoreduction is evolutionary imprinted in all LOV domains, while efficient pT is needed to stabilize the neutral semiquinone radical.

## Introduction

**Figure 1:**
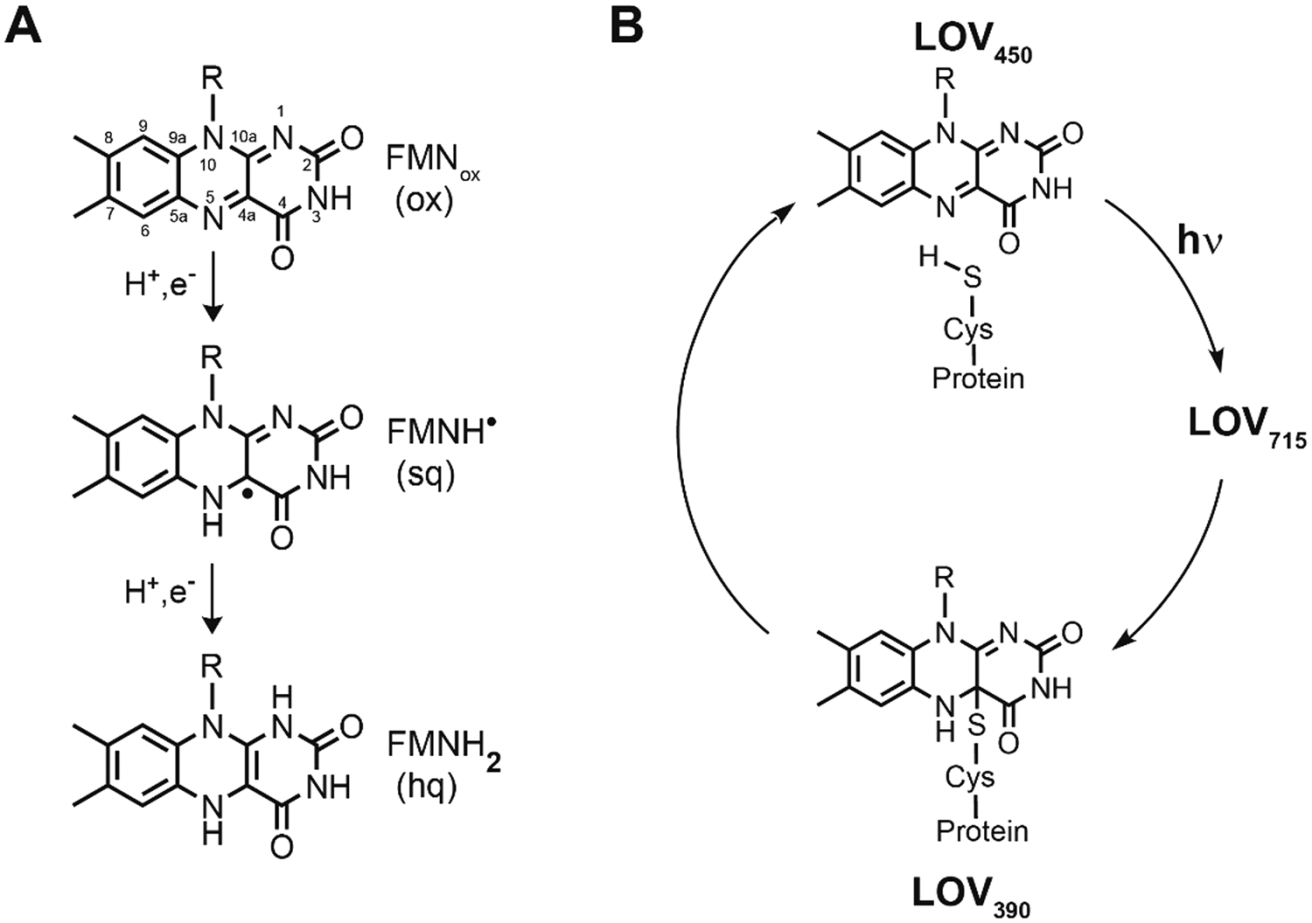
Structures of the redox states of FMN (**A**): the oxidized FMN (ox), the one-electron reduced neutral semiquinone radical FMNH^●^ (sq) and the fully reduced FMNH_2_ (hq). Schematic of the LOV domain photocycle (**B**): Upon blue-light absorption in the dark state (LOV450), the excited singlet state of FMN is formed. After conversion to the excited triplet state (LOV715), a covalent flavin-cysteine adduct is formed (LOV390), which recovers back to LOV450 in the dark.

Flavin-binding proteins are ubiquitously distributed throughout all kingdoms of life, playing important roles either as redox catalysts^1,2^ or molecular sensors for redox potential, oxygen or blue light^3–5^. The latter function is intricately linked to the oxidation state and photochemistry of the bound flavin molecule. The flavin molecule itself can exist in three redox states, oxidized (quinone, ox), one-electron reduced (semiquinone, sq) and fully reduced (hydroquinone, hq) (Fig. 1A). In particular, the oxidized form of flavins strongly absorbs light in the blue region of the spectrum thereby allowing the functional response of the photoreceptor protein^5^. Flavin-binding blue-light photoreceptors can be classified according to structure and photochemistry into three main groups: i) sensors of blue-light using flavin adenine dinucleotide (FAD) (BLUF photoreceptors), ii) cryptochromes (CRYs) and iii) light, oxygen, voltage (LOV) photoreceptors^3^. In each of those families, photoreceptor proteins undergo distinct photochemical reactions upon blue-light exposure, which in turn are directly linked to conformational changes in the protein, allowing for blue-light dependent physiological responses of the respective host^3,5,6^.

LOV photoreceptors represent an architecturally diverse photoreceptor family^7^, regulating a myriad of different responses in bacteria, fungi and plants^3,5,6,8–11^. In the dark the LOV domain (i.e. the sensory domain of all LOV photoreceptors) non-covalently binds oxidized flavin mononucleotide (FMN_ox_) (or in some cases the oxidized form of FAD or Riboflavin)^3,12^ resulting in an absorption maximum at 450 nm (LOV_450_; Fig. 1B). Upon illumination with blue light the excited singlet state of FMN is formed, which undergoes intersystem crossing to the excited triplet state (LOV715; Fig. 1B). Subsequently, a covalent bond between a strictly conserved cysteine residue in the vicinity of the flavin chromophore and the C4a atom of the FMN isoalloxazine ring is formed^3,13^, resulting in the loss of absorbance at 450 nm and a concomitant increase in absorbance at 390 nm (LOV_390_; Fig. 1B). The exact mechanism of adduct formation is still under debate, however a radical-pair mechanism is thought to be most likely^3,14–16^, following the idea that formation of the triplet state induces electron and proton transfer reactions from the conserved cysteine resulting in the rapid reduction of FMN by the thiol group of the cysteine to form a neutral radical pair (FMNH^●^/Cys-S^●^). In turn, the radical pair recombines to form the FMN-cysteinyl-thiol adduct with the N5 atom of the flavin being protonated (LOV390; Fig. 1B). In particular, the built up of the radical intermediate has not been proven unequivocally for a wild-type LOV protein^14,15^. In the dark, the flavin:cysteine covalent bond is broken spontaneously and the N5 atom of the FMN chromophore is deprotonated to conclude the photocycle. Thus, photocycling in LOV domains depends on both the formation of a covalent flavin-cysteine adduct and the protonation of the flavin N5 atom.

Recently, Yee *et al*. showed that the functional light-dependent responses of two LOV photoreceptors, namely of *Neurospora* VVD and of the artificial LOV histidine kinase YF1, do not strictly depend on the presence of the conserved cysteine^17^. The authors demonstrated that the accumulation of the neutral flavin semiquinone radical FMNH^●^, and hence flavin N5 protonation, is sufficient to elicit a functional response in photoreceptor variants containing an alanine instead of the photoactive cysteine, although with reduced magnitude compared to the respective wild-type proteins^17^. Similar effects have been reported for plant (*Arabidopsis thaliana*) and algal (*Chlamydomonas reinhardtii*) phototropins, in which the LOV1, the LOV2 or both LOV domains were inactivated by replacing the respective photoactive cysteine against alanine^18–20^.

Interestingly, different LOV variants devoid of the photoactive cysteine show different propensity towards photoreduction. While apparently, VVD-C108A and YF1-C62A can effectively be photoreduced in the presence of oxygen, although quite high light intensities were needed (VVD-C108A, 30 mW 488 nm laser; YF1-C62A, 50 mW cm^−2^ 455 nm light), other LOV variants can only be photoreduced under anaerobic conditions and/or in the presence of external sacrificial electron donors such as ethylenediaminetetraacetic acid (EDTA)^21–24^. Thus, for LOV variants devoid of the photoactive cysteine it is currently unclear which (structural) factors determine photoreduction efficacy. Moreover, the residues which act as proton/electron donor in the photoreduction process remain largely elusive.

To address this issue we set out to reengineer efficient photoreduction into a LOV protein variant which shows no propensity towards photoreduction in the presence of oxygen. Based on this selection criterion, we chose the very photostable LOV-based fluorescent protein iLOV as engineering target, which is derived from the *A. thaliana* phototropin-2 (AtPhot2) LOV2 domain^25^. To introduce a potential proton donor (other than cysteine) we substituted a conserved glutamine residue (Q489) in the vicinity of the flavin chromophore for aspartic acid. While the parent iLOV protein cannot be photoreduced in the presence or absence of oxygen and the addition of excess amounts of EDTA is necessary for photoreduction, iLOV-Q489D is efficiently photoreduced in the presence of oxygen. The observed photoreduction pH dependency of iLOV-Q489D, as well as modelling studies, suggest that the introduced Asp acts as proton donor for the photoreduction process. Transient absorption (TA) spectroscopic measurements reveal efficient formation of a very long-lived FMNH^●^ state indicative for the presence of a stabilizing counter radical in the protein. Analysis of the TA data provides evidence that Trp^●^ represents the counter radical in iLOV-Q489D. The presence of an FMN:protein radical pair was further corroborated by electron paramagnetic resonance (EPR) spectroscopy. The sole mutation of individual Tyr and Trp residues (as the potential electron donors) for phenylalanine in iLOV-Q489D did not abolish photoreduction but revealed differences in photoreduction yield and FMNH^●^ formation kinetics, which suggests that more than one of those residues can transfer an electron to the excited flavin chromophore, either by forming a Tyr/Trp cascade similar to cryptochromes^3,26^ or by independently acting as electron donor for FMN photoreduction.

## Results

### Target protein selection and design rationale

LOV proteins in which the photoactive cysteine has been substituted for alanine cannot undergo adduct formation and thus show an intense cyan-green fluorescence resulting from excitation of the bound flavin chromophore^13,25,27^. Those LOV-based fluorescent proteins (LOV-FP), also called flavin-binding fluorescent proteins (FbFPs), represent a promising alternative to reporter proteins of the green fluorescent protein (GFP) family^25,27^. Compared to GFP and related proteins, LOV-FPs often show reduced photostability^28,29^. One potential cause for photobleaching is the photoreduction of FMN to the neutral semiquinone radical FMNH^●^. Thus, a photostable LOV-FP should principally show little or no photoreduction in the presence of oxygen. Dictated by this selection rule we decided to use the iLOV protein^25^, a LOV-based FP with high photostability, as engineering target. The iLOV protein was derived from the LOV2 domain of *A. thaliana* phototropin-2 (AtPhot2) by substitution of the photoactive cysteine for alanine (C426A; AtPhot2 numbering) and several rounds of DNA shuffling^25^. To achieve efficient FMN photoreduction to yield FMNH^●^, both an electron and a proton donor are needed. Hereby, the respective electron or proton donating amino acids need to be placed at suitable position in close proximity to the FMN chromophore without disturbing the protein structure. Moreover, they should adopt a conformation that facilitates the transfer. Recently, a study on the LOV1 domain of *C. reinhardtii* was published, in which a Tyr was inserted as a potential electron donor in close proximity to the FMN chromophore^30^. In this CrLOV1-F41Y mutant, photoreduction of the FMN is favored over adduct formation although the reactive Cys is present. We therefore here decided to use an alternative approach, reengineering proton transfer (pT) while utilizing potential naturally existing electron donors in iLOV for photoreduction. To this end, we introduced an aspartate residue as potential proton donor in close vicinity of the flavin chromophore. Apart from the photoactive cysteine, or the introduced alanine in case of LOV-FPs such as iLOV, only a highly conserved glutamine residue (Q489 in iLOV) is in a suitable position to enable proton transfer. According to the X-ray structure and our previous MD simulations^31^ this amino acid forms a hydrogen bond with the FMN-O4 atom and is in close proximity of the FMN-N5 atom (Figure S1 in SI).

### Illumination of iLOV-Q489D results in effective formation of FMNH^●^ under aerobic conditions

**Figure 2:**
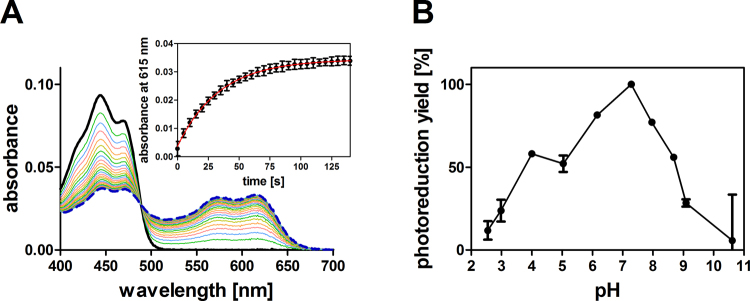
Spectral changes associated with photoreduction of iLOV-Q489D at pH 7.2 (**A**). The dark-adapted sample (solid black line) was illuminated with blue light (0.13 mW cm^−2^) in 5 seconds time increments and sequential spectra were recorded (rainbow coloring) until no further spectral changes did occur (dashed blue line). The last spectrum corresponds to an illumination time of 150 seconds. The inset depicts the time trace of iLOV-Q489D photoreduction derived from the rise in absorbance at 615 nm. Experimental data was fit using a single-exponential decay function (red line) and proceeds with a photoreduction lifetime of τ_Sq_ = 25.7 ± 0.2 s. (**B**) pH dependence of iLOV-Q489D photoreduction yield. iLOV-Q489D photoreduction, at a given pH, was quantified from UV/Vis spectra by determining the rise in absorbance at 615 nm after 150 seconds illumination. The photoreduction yield is here defined as the relative yield of FMNH^●^. Photoreduction yields are expressed relative to the maximum value (100%) at pH 7.2. Error bars correspond to the standard deviation of the mean derived from three independent measurements.

As expected for a photostable LOV-FP, the parent iLOV protein does not show any detectable blue-light (0.13 mW cm^−2^) dependent photoreduction under aerobic or anaerobic conditions (Figure S2A and B), and the addition of excess amounts of EDTA as electron donor under anaerobic conditions is necessary to photoreduce FMN to FMNH^●^ (Figure S2C). In contrast, iLOV-Q489D is readily photoreduced even under aerobic conditions (Fig. 2A).

Hereby, the illumination of dark-adapted iLOV-Q489D results in the loss of the absorbance band at around 450 nm, which corresponds to fully-oxidized FMN_ox_ (solid black line). Concomitantly a new species with broad red-shifted absorbance with maxima at around 576 nm and 615 nm (dashed blue line) is formed, corresponding to FMNH^●^^22^. Photoreduction is complete within 150 seconds and proceeds with a lifetime of τ_Sq_ = 25.7 ± 0.2 s (Fig. 2A, inset). A more detailed analysis of the spectral changes can be found in the SI (Figures S5–S8). Here, i.e. singular value decomposition of a dataset recorded from 250 nm to 650 nm at pH 7.2 provides clear evidence that only two components (FMN_ox_, FMNH^●^) significantly contribute to the spectra, thus essentially ruling out the formation of significant amounts of the anionic semiquinone radical or the hydroquinone. To rule out that the observed photoreduction of iLOV-Q489D is caused by reduced oxygen access to the active site compared to parental iLOV, we determined the efficacy of the reoxidation process after photoreduction for both iLOV-Q489D and parental iLOV (Figure S2D). Hereby, reoxidation proceeds similarly fast in iLOV-Q489D (τ_FMNox_ = 1502 ± 27 s) and parental iLOV (τ_FMNox_ = 1376 ± 520 s), suggesting that reduced oxygen access is not the cause for the observed efficient photoreduction of iLOV-Q489D.

To evaluate the pH dependence of the iLOV-Q489D photoreduction process, we carried out identical measurements in a pH range between pH 2.6 and 10.6 (Fig. 2B). The spectral changes associated with illumination of iLOV-Q489D at pH 2.6 to pH 10.6 are summarized in Figure S3. Using this data, the photoreduction yield, defined here as the relative yield of the FMNH^●^, at a given pH was quantified from UV/Vis spectra by determining the rise in absorbance at 615 nm due to illumination (Fig. 2B). The photoreduction yield is low at both acidic (<pH 3) and basic (>pH 9) pH values and shows a maximum at pH 7.2. While the very low photoreduction yields observed below pH 4.0 can readily be explained by pH-dependent unfolding of iLOV-Q489D which results in the release of the flavin chromophore from the protein thereby abolishing photoreduction, iLOV-Q489D is stable at high basic pH values (see SI, Figure S4). Thus, the low photoreduction yields at basic pH values cannot be attributed to unfolding of the protein. More detailed analyses of the pH-dependent effects can be found in the SI (Figures S4–S8).

In conclusion, when the pH stability (Figure S4) and photodamage (Figure S7) of iLOV-Q489D is taken into account, the observed pH dependence of the iLOV-Q489D photoreduction process appears to be linked to a proton transfer process which is most efficient at neutral pH values. Hence, the side chain of the newly introduced Asp489 in iLOV-Q489D must be protonated at neutral pH values. Compared to free aspartate (pK_a_ = 3.9) the pK_a_ value of Asp489 must thus be substantially shifted towards more neutral pH values which could be caused by the protein environment^32–34^. To account for this possibility we computationally introduced the Q489D mutation in the parent iLOV structure (PDB ID: 4EES) and predicted the pK_a_ of the introduced Asp by using the PROPKA 3.1 program^35^. In line with the experimental data, PROPKA predicts a pK_a_ of 6.28 for D489 iLOV-Q489D, which, along with the observed lack of photoreduction in parental iLOV, corroborates the assumption that D489 in iLOV-Q489D acts as proton donor. Additional experimental data and molecular dynamic simulations that strengthen this notion are presented in the Figure S8.

### Transient absorption spectroscopy hints at the formation of a stable FMN:protein radical pair in iLOV-Q498D

**Figure 3:**
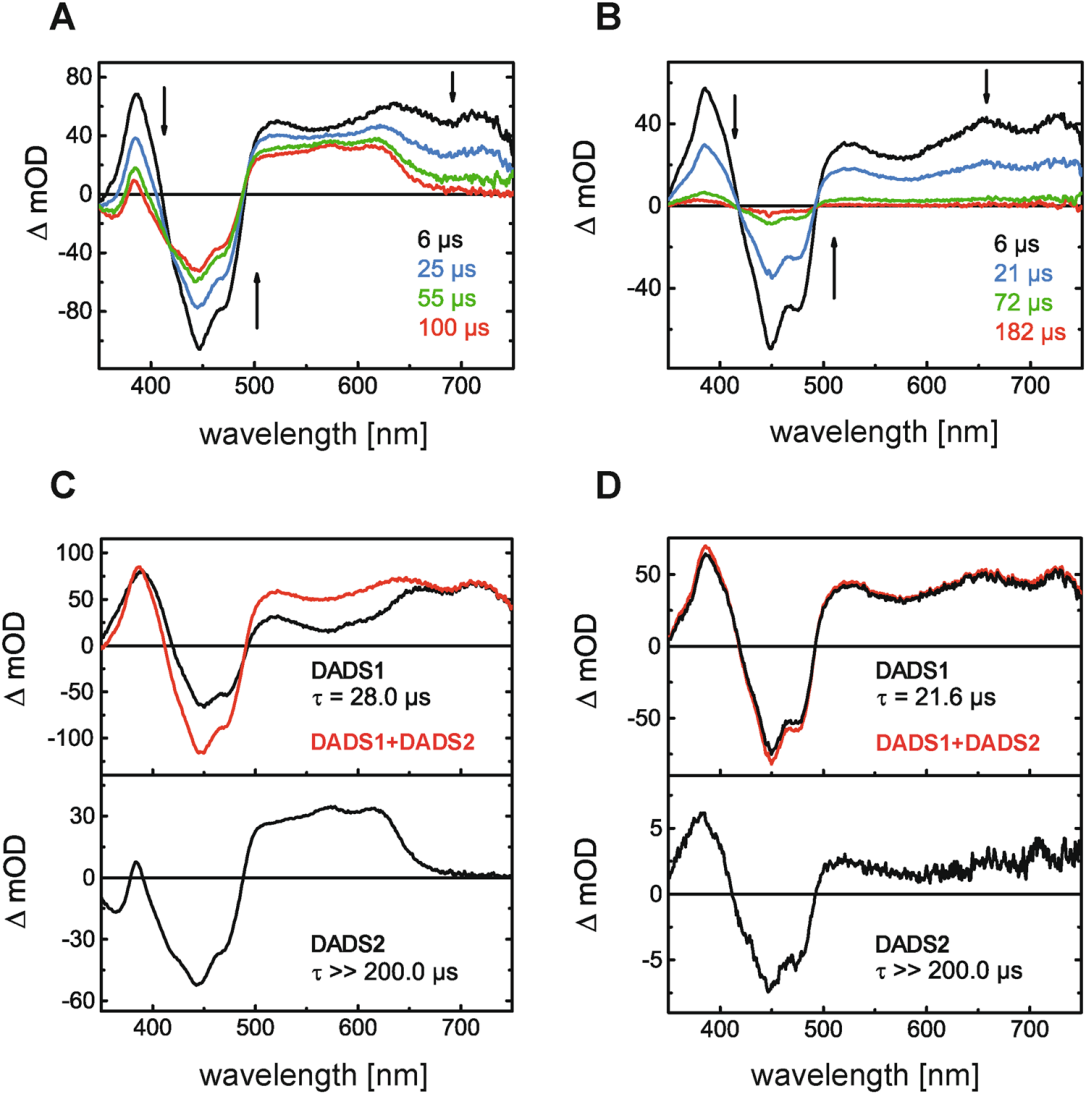
Difference absorption spectra of iLOV-Q489D (**A**) and parental iLOV (**B**) extracted from TA datasets at indicated times. TA was measured on a 200 μs time window. DADS of iLOV-Q489D (**C**) and parental iLOV (**D**) resulting from global lifetime analysis. DADS1 decays with a time constant of 28.0 μs in the case of iLOV-Q489D and 21.6 μs for parental iLOV. The second DADS are non-decaying within the measured time window in both samples. The sum of both DADS (red lines in **C** and **D**) represents the situation of the systems at t_0_, i.e. immediately after the ns laser excitation pulse.

**Figure 4:**
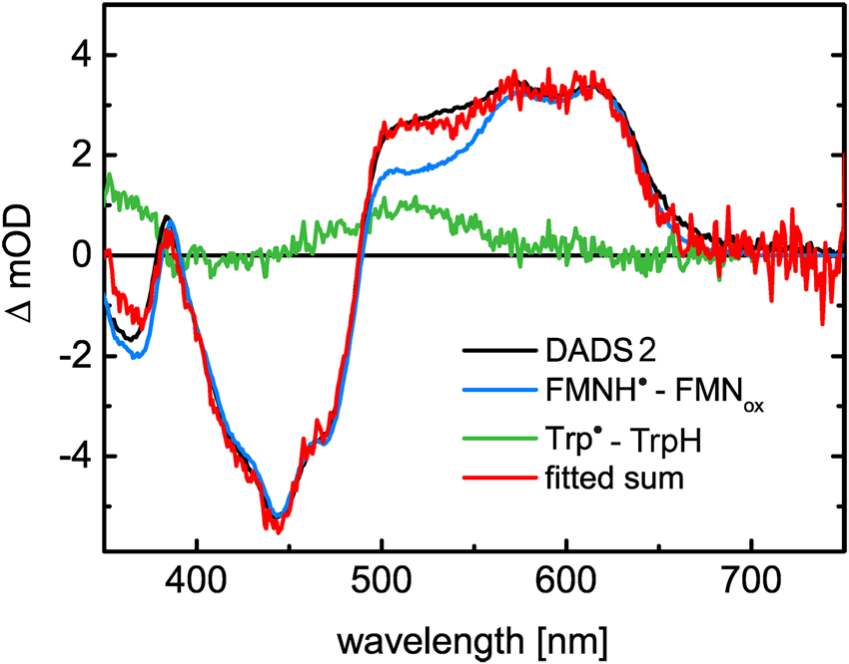
DADS2 of iLOV-Q489D (black line and Fig. 3, (**C**)) shows typical spectral features of FMNH^●^ with a peak around 390 nm and a fine structured, broad absorption band between 490 nm and 670 nm. For direct comparison, the difference spectrum of FMNH^●^-FMN_ox_, obtained in the steady state UV/Vis spectra, is shown (blue line). To account for the differences in the range of 490 nm to 550 nm, the contribution of Trp^●^ (green line) is necessary for spectral fitting of DADS2.

In order to gain insight into the mechanistic details upon blue-light excitation, transient absorption (TA) spectroscopy was performed with iLOV-Q489D and parental iLOV. Figure 3A and B show the difference absorption spectra of iLOV-Q489D and parental iLOV extracted from the 2-dimensional TA data measured on a 200 µs time window. The negative absorption band in the range of 410 nm–490 nm can be assigned to the ground-state bleach of FMN_ox_. Furthermore, a positive fine-structured absorption band above 650 nm can be observed, which decays completely within 100 µs. The positive peak between 360 nm and 410 nm decreases simultaneously within this time range. In the case of iLOV-Q489D (Fig. 3A), after 100 µs the transient absorption signal stays constant within the measured time window, with a significant positive contribution between 490 nm and 650 nm and a minor absorption band around 385 nm. In contrast to this, almost no transient absorption signals can be detected for parental iLOV after 182 µs (Fig. 3B). To gain further insight into the underlying processes, global lifetime analysis was performed resulting in the decay associated difference spectra (DADS) of iLOV-Q489D (Fig. 3C) and parental iLOV (Fig. 3D). In both cases two contributing species were sufficient to fit the data. The first DADS, DADS1, decays with a rate constant of 28.0 µs (iLOV-Q489D) and 21.6 µs (parental iLOV), respectively. Next to the negative absorption signal of the ground state bleach of FMN_ox_, the DADS1 exhibit positive bands between 350 nm and above 495 nm. By comparison with published flavin spectra^36^, these DADS can be associated with the triplet state absorption of FMN. The DADS2 of iLOV-Q489D and parental iLOV are both non-decaying within the measured time window, and both spectra differ strongly. DADS2 of parental iLOV is very weak and looks highly similar to the triplet decay spectrum represented by DADS1, indicating a biphasic triplet decay. The slower decaying component in DADS2 has less than 5% of the amplitude of DADS1. This might indicate a second quenching channel in parental iLOV. DADS2 of iLOV-Q489D, on the other hand, exhibits a strongly positive, fine-structured absorption band between 490 nm and 650 nm and a smaller peak around 385 nm, as well as the negative absorption band of the ground-state bleach of FMN_ox_. The broad, vibronic fine-structured absorption band above 500 nm is a characteristic feature of FMNH^●^^36–38^ indicating that electron transfer occurs in iLOV-Q489D. For direct comparison, the difference spectrum of FMNH^●^ minus FMN_ox_, derived from steady-state UV/Vis spectra of iLOV-Q489D, is shown together with DADS2 in Fig. 4. Although the main features are the same, the two spectra differ significantly in the range of 492 nm to 560 nm.

Thus, apparently an additional species contributes to DADS2. The efficient formation of FMNH^●^ in iLOV-Q489D originates from an electron transfer reaction, suggesting that the third component in DADS2 is due to the radical species produced by the electron donor, most likely one of the amino acids of iLOV-Q489D. Natural candidates are Trp and Tyr. The neutral tyrosyl radical, TyrO^●^, is known to have solely a narrow absorption peak around 405 nm in the visible range^39,40^, while the corresponding Trp^●^ has a distinct absorption between 440 nm and 580 nm^41,42^, indicating that Trp^●^ is more likely the counter-radical of FMNH^●^. TrpH^●+^ absorbs as well in the visible range with a broad absorption band between 450 and 650 nm exhibiting a maximum at 560 nm and additionally at 335 nm^42^, but since the neutral FMNH^●^ is formed, the corresponding radical species of the electron donor is most likely also present in its neutral state. As further support for the contribution of Trp^●^ to DADS2 of iLOV-Q489D, we generated a reference spectrum of the tryptophanyl radical in solution at pH 8.0 (see Figure S9 in SI, inset). DADS2 of iLOV-Q489D could be well represented as a linear combination of the difference spectrum FMNH^●^-FMN_ox_ and the Trp^●^ reference spectrum. The best fit is shown as the red line in Fig. 4. This strongly suggests that the electron donor responsible for the efficient formation of FMNH^●^ in iLOV-Q489D is W467, the only Trp of the protein.

### FMNH^●^ formation occurs from the excited triplet state in iLOV-Q489D

Global lifetime analysis provides evidence for the formation of the triplet state in both iLOV variants. It is noteworthy that the spectral shape of the DADS1 in Fig. 3C and D differs significantly between 525 nm and 650 nm. When the lifetimes obtained by global lifetime analysis differ considerably (i.e. by more than a factor of 10), each DADS represents the spectrum of the species decaying with this lifetime minus the spectrum of the evolving species. In the case of DADS1 of iLOV-Q489D the decaying species is the excited triplet state of FMN, and the products are the FMN ground state, FMN_ox_, and -under the assumption that the observed flavin radical is formed via the triplet state- FMNH^●^. As support for the hypothesis of an electron transfer reaction via the excited triplet state of flavin in iLOV-Q489D, the sum of all DADS of parental iLOV and iLOV-Q489D are shown for comparison in Fig. 3C and D (red lines). The sum of DADS1 and DADS2 represents the initial situation of the iLOV proteins at t_0_ immediately after the laser excitation pulse of 10 ns duration, and before any decay processes on the µs-timescale occur. The spectra are identical for parental iLOV and iLOV-Q489D (direct comparison not shown). Since no radical species could be observed in the case of parental iLOV, the t_0_ spectrum represents the pure triplet spectrum of FMN in both iLOV variants. We conclude that the observed FMNH^●^ was not already formed at t_0_ in iLOV-Q489D. This rules out FMNH^●^ formation from the excited singlet state. Further support is given to this hypothesis by the fluorescence decay time τ_fl_ = 5.0 ns of the S_1_ state of iLOV-Q489D. The same value has been reported for parental iLOV^43^. This fluorescence lifetime is characteristic for FMN_ox_ in flavoproteins that do not undergo photoinduced electron transfer from the excited singlet state.

### Electron paramagnetic resonance (EPR) spectroscopy reveals the presence of a stable radical pair in iLOV-Q489D

**Figure 5:**
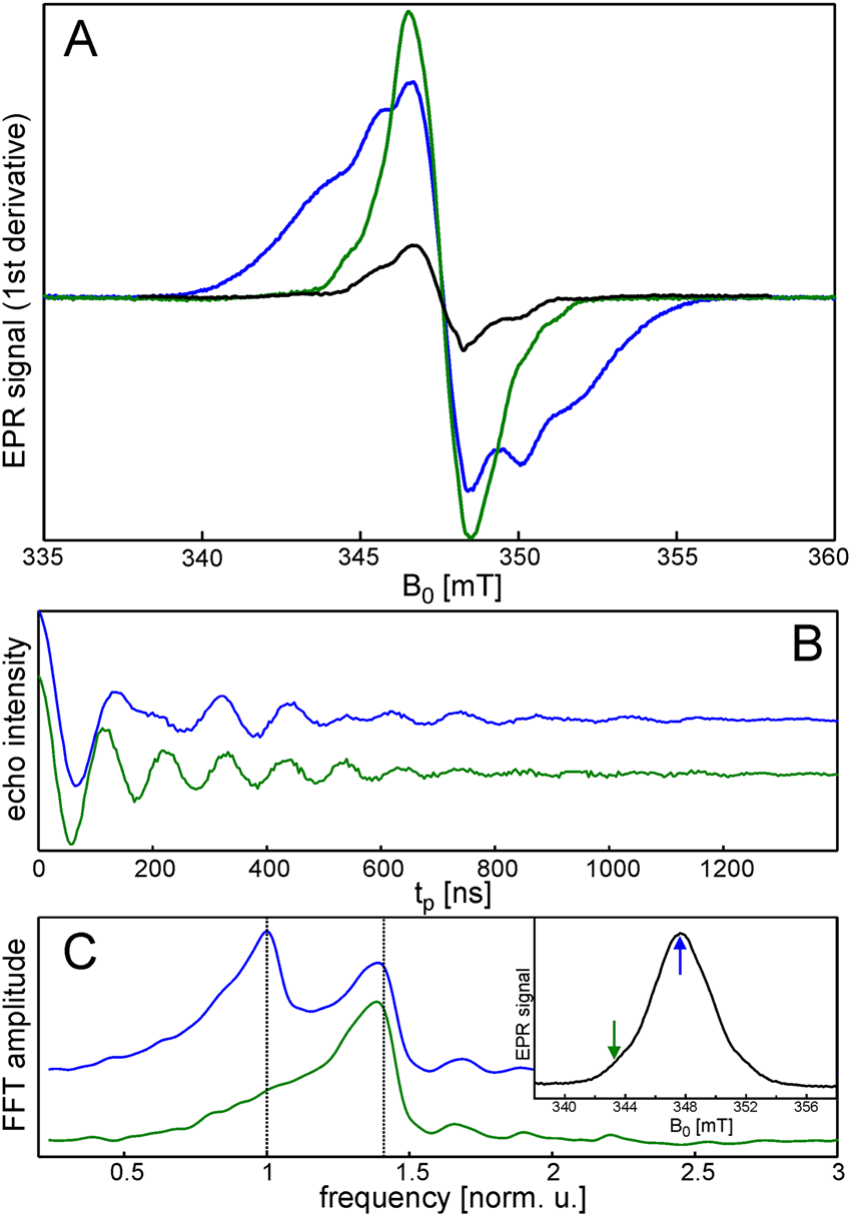
(**A**) X-band continuous wave (cw) EPR spectra of parental iLOV (black trace) and iLOV-Q489D (blue trace) recorded after 20 min illumination of the dark adapted samples. The green trace shows the EPR spectrum of illuminated iLOV-Q489D after annealing for 10 min at 240 K. All spectra were acquired at 120 K. (**B**) Electron spin echo nutation time traces and (**C**) their Fourier transforms for the illuminated iLOV-Q489D sample recorded at the magnetic field positions marked by arrows in the inset panel (**C**) which shows field-swept echo-detected EPR spectrum at 120 K. The dashed lines in (**C**) mark the frequency positions of FFT amplitude maxima. The frequency axis is normalized to the frequency of the low frequency peak in the upper trace.

Figure 5 shows X-band continuous-wave (cw) EPR spectra of parental iLOV (black trace) and iLOV-Q489D (blue trace) illuminated with a pulsed 450 nm laser for 20 min at 120 K. More prolonged illumination does not lead to significant changes in the EPR spectra. The EPR spectrum of parental iLOV is composed of a symmetrical line positioned around g = 2.0034 with partially resolved hyperfine structure. This spectrum is virtually identical to that of the neutral semiquinone FAD radical in *Escherichia coli* DNA photolyase reported previously^44^. In contrast, the EPR spectrum of iLOV-Q489D spreads over a larger frequency range (~15 mT). It is thus much broader than EPR spectra of individual radicals expected in the system, i.e. FMNH^●^^44^, tyrosine^45^ or tryptophan^45–48^ radicals. The nature of this signal was revealed using the electron spin echo nutation experiment. Here, a preparation microwave pulse of variable duration is inserted in the pulse sequence before the Hahn echo detection block, and the oscillation (nutation) of the echo signal is monitored as a function of the preparation pulse length (*t*_p_). The frequency of the EPR signal oscillation with increasing preparation pulse length (nutation frequency, *ν*_nut_) is related to the spin quantum numbers (*S*, m_s_) of the excited EPR transition^49,50^ according to the equation:

1 $${v}_{nut}=\frac{\alpha \,\cdot {\omega }_{1}}{2\pi };\,\alpha =\sqrt{S(S+1)-{m}_{s}({m}_{s}\pm 1)}$$

where *ν*_nut_ is the nutation linear frequency, *ω*_1_ is the microwave field strength in angular frequency units, and the *α* factors describing the transitions in *S* = ½ and *S* = 1 spin manifolds are α = 1 and α = $\sqrt{2}$, respectively. The nutation curves recorded at two magnetic field positions for iLOV-Q489D are shown in Fig. 5B.

Their Fourier transforms are depicted in Fig. 5C. At the low field position the nutation spectrum demonstrates a single peak whereas in the center it is composed of two well resolved peaks with a frequency ratio of 1.4 (see Fig. 5C). Thus, the broad spectral contribution is assigned to a triplet *T* (*S* = 1) state species. The spectral intensity in the center stems as expected from doublet *D* (*S* = ½) radical species (see Figure S10). The simulation of the EPR spectrum using overall inhomogeneous spectral line width of Gaussian shape of 3 mT yields the fine structure parameter of |D| = 4 ± 1 mT and |E| = 0 (see Figure S11). The parameter D is directly connected to the distance r between two spins:

2 $$D=\frac{2.79}{{r}^{3}}T\cdot {{\rm{\AA }}}^{3}$$

assuming g-values of paired radicals to be equal to those of the free electron g_e_. Thus, the estimated D value corresponds to 9 ± 1 Å electron-electron distance in point dipole approximation, i.e. neglecting spin distribution over the radical structures. This estimate gives the shortest possible distance, because neither the real spectral shapes of individual radicals nor the spin density distribution can be taken into account in the analysis. In order to obtain additional information about possible electron transfer pathways, annealing experiments were performed. The EPR spectrum of iLOV-Q489D was continuously monitored while the sample temperature was increased from 120 K up to 200 K (about the glass transition temperature of water/glycerol solution), no changes were observed. Above 200 K the spectrum changes, the broad contribution continuously disappeared in favor of a narrow line at g = 2.0034. The spectrum recorded at 120 K after sample equilibration at 240 K for 10 minutes is shown in Fig. 5A (green line). This spectrum mostly contains the contribution from FMNH^●^. It is in good agreement with one recorded after room-temperature illumination and subsequent trap freezing in liquid nitrogen (data not shown). Taken together, EPR spectroscopy performed at low temperature corroborates the presence of a flavin:protein radical pair, which was also observed at room temperature in transient absorption (TA) measurements of iLOV-Q489D. While no radical formation is observed for parental iLOV in room-temperature TA measurements, FMNH^●^ is detected at low temperatures by EPR, although with a much lower amplitude.

### Mutational analysis of the electron transfer pathway in iLOV-Q489D suggests the involvement of multiple Tyr/Trp residues

As potential electron donors for the photoreduction process, and hence candidates for the amino acid constituting the counter protein radical, iLOV-Q489D possesses three tyrosines (Y416, Y459 and Y484) as well as the above mentioned tryptophan (W467). In the X-ray structure of parental iLOV (Figure S12) they are located at an edge-to-edge distance of 9.9 Å (Y416), 9.1 Å (Y459), 10.2 Å (Y484) and 10.8 Å (W467) from the FMN molecule (for details see Table S1). Hence, based on the above presented EPR experiments, which revealed a minimum electron-electron distance of 9 ± 1 Å between the flavin radical and the protein radical, none of those residues can be excluded as potential electron donor. To address this issue, we generated variants of iLOV-Q489D in which the Tyr and Trp residues were substituted for phenylalanine. The corresponding double mutants were expressed, purified and characterized in an identical manner as parental iLOV and iLOV-Q489D. Surprisingly, all double mutants showed photoreduction in the presence of oxygen (Figure S13). The detailed analysis of the photoreduction yield and FMNH^●^ formation kinetics is given in Table 1. Among the double mutants, only iLOV-Q489D/W467F showed a significantly reduced photoreduction yield (89 ± 0.5%) relative to iLOV-Q489D. All other double mutants showed photoreduction yields that were, within the error of the measurement, identical to the one observed for iLOV-Q489D. Compared to iLOV-Q489D all variants showed significantly faster FMNH^●^ formation (see τ_Sq_ in Table 1), while the reoxidation process was not affected (Figure S13).

**Table 1:** Photoreduction yields and FMNH^●^ formation kinetics of iLOV-Q489D, iLOV-Q489D/Y416F, iLOV-Q489D/Y459F, iLOV-Q489D/Y484F and iLOV-Q489D/W467F.

Protein	Photoreduction yield [%]^§$^	τ_Sq_ [s]^$^
iLOV-Q489D	100 ± 6	25.7 ± 0.2
iLOV-Q489D/Y416F	101 ± 1(NS)	13.2 ± 0.8 (***)
iLOV-Q489D/Y459F	103 ± 2 (NS)	5.6 ± 0.1 (***)
iLOV-Q489D/Y484F	101 ± 2 (NS)	20.8 ± 0.6 (***)
iLOV-Q489D/W467F	89 ± 0.5 (**)	11.5 ± 0.4 (***)

^§^Photoreduction yield relative to the yield of iLOV-Q489D (100%). Photoreduction yield defined as the relative yield of the neutral semiquinone radical FMNH^●^. ^$^Values represent the mean and standard deviation of the mean derived from three independent measurements. Statistical significance (in brackets) of the observed differences was tested by One-Way ANOVA and Dunnett’s Post Test (P < 0.01) for comparison against iLOV-Q489D. NS: Not Significant.

**Figure 6:**
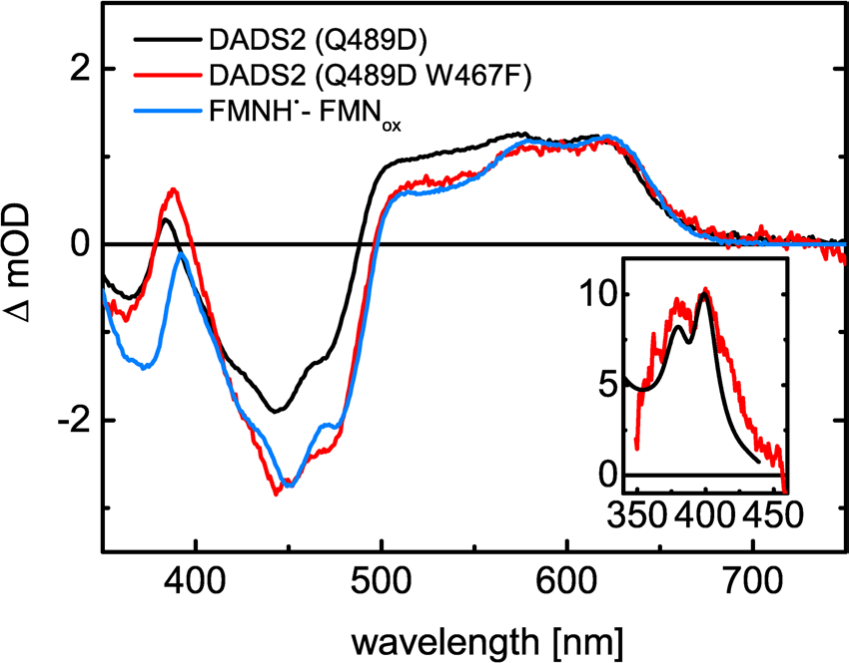
Comparison of the DADS2 of iLOV-Q489D (black line) and iLOV-Q489D/W467F (red line) derived from global analysis of the TA data. The spectra are scaled to same value at 620 nm. The reference spectrum of FMNH^●^-FMN_ox_ (blue line) is in good agreement with the DADS2 of iLOV-Q489D/W467F above 420 nm. Inset: Spectrum obtained by subtraction of the reference spectrum FMNH^●^ -FMN_ox_ from the DADS2 of iLOV-Q489D/W467F corresponding to TyrO^●^ (red line). For comparison, a reference spectrum of TyrO^● 51^ is included (black line), blues shifted by 10 nm.

Since iLOV-Q489D/W467F shows efficient photoreduction although the electron donor Trp, identified by TA, has been substituted for Phe, we additionally performed TA measurements of this variant. Global lifetime analysis of the 2D TA data of iLOV-Q489D/W467F results in two DADS (Figure S14) similar to the spectra we observed in the case of iLOV-Q489D: DADS1 can be associated with the excited triplet state of FMN and decays with τ = 108 µs. This is by a factor of four slower compared to iLOV-Q489D and parental iLOV. DADS2 of iLOV-Q489D/W467F, on the other hand, is characterized by the typical absorbance of FMNH^●^, which is formed via the excited triplet state as indicated by the t_0_ spectrum. Since iLOV-Q489D/W467F is lacking the Trp, which we identified to serve as the counter radical of FMNH^●^ in iLOV-Q489D, the DADS2 of both iLOV variants are compared in Fig. 6. Both DADS2 are scaled to the same value at 620 nm. At this wavelength only FMNH^●^ absorbs in iLOV-Q489D and Q489D/W467F. When comparing both spectra, it can be clearly seen that DADS2 of iLOV-Q489D (black line) has more positive absorption in the range of Trp^●^ absorption from 490 nm to 575 nm than DADS2 of iLOV-Q489D/W467F (red line). However, it should be noted that the amplitude of the ground-state bleach differs between the two iLOV spectra. This is due to positive absorption of Trp^●^ until 440 nm (see Fig. 4) and a certain fraction of irreversible photodamage of iLOV-Q489D/W467F during the TA measurement. When comparing DADS2 of iLOV-Q489D/W467F with the difference absorption spectra of FMNH^●^, obtained by linear combination of the steady-state spectra of FMNH^●^ and FMN_ox_, both spectra are in good agreement above 420 nm. Subtraction of the difference absorption spectrum of FMNH^●^ from DADS2 of iLOV-Q489D/W467F yields the spectrum shown in the inset in Fig. 6. The typical narrow absorption band at 400 nm with a shoulder at 380 nm can be assigned to the neutral radical of tyrosine, TyrO^●^, blue-shifted by 10 nm compared to previously reported spectra^39,40,51^. The observation of TyrO^●^ demonstrates that one of the tyrosines of iLOV-Q489D/W467F substitutes W467 as the counter-radical of FMNH^●^.

## Discussion

A recurrent theme observed in various flavin-binding photoreceptors is the light-driven photoreduction of oxidized flavin via electron transfer (eT) and subsequent proton transfer (pT) reactions to yield the neutral semiquinone radical FMNH^●^^3^. Very recently, it was shown for different LOV photoreceptors that FMNH^●^ formation (photoreduction) in LOV variants lacking the photoactive cysteine residue, suffices to elicit a functional response of the photoreceptor^17^. Hereby, recent simulations suggested, that the initial structural response of LOV domains, i.e. the glutamine amide flip (i.e. Q182 in VVD) occurs in response to either adduct formation or reduction of the isoalloxazine ring to the FMNH^●^, both of which involve N5 protonation. In different cysteine-less LOV proteins, the propensity for FMN photoreduction seems to vary widely and, most importantly, the residues involved in eT and pT reactions remain largely elusive, although a [FMN^●−…^Trp^●+^] radical pair has been identified before in cysteine-less LOV domains by NMR-based photochemically induced dynamic nuclear polarization (photo-CIDNP) studies^52–55^. Though, to the best of our knowledge, a stable [FMNH^●…^Trp^●^] radical pair has so far not been observed directly. We therefore set out to reengineer the photochemistry of the flavin chromophore in the photostable LOV-based fluorescent protein iLOV, which lacks the photoactive cysteine. The photostability of the protein hereby suggests that iLOV is not prone to photoreduction in the presence of oxygen, rendering it the ideal target protein for studying and introducing eT and pT reactions. The aforementioned detection of a [FMN^●−…^ Trp^●+^] radical pair in cysteine-less LOV domains via photo-CIDNP^52–55^ suggests that eT pathway(s) are naturally present in these proteins. Given the conserved nature of the flavin-binding pocket in LOV proteins, we reasoned that the most important step for stabilizing FMNH^●^ in iLOV would be the introduction of a suitable proton donor. We therefore introduced an Asp residue at the position of a highly conserved Gln (Q489) which in parental iLOV is forming H-bonds to the FMN chromophore. The steady-state UV/Vis spectra show that, while parental iLOV cannot be photoreduced in the presence or absence of oxygen (Figure S2) without the addition of an external sacrificial electron donor (Figure S2), iLOV-Q489D is efficiently photoreduced in the presence of oxygen rapidly accumulating FMNH^●^ during illumination (Fig. 2). The observed pH dependence of the photoreduction process as well as modelling studies suggest that the newly introduced Asp is (at least partially) protonated at neutral pH values and hence can serve as proton donor.

Our EPR studies provide evidence for the formation of a stable protein:FMN radical pair in iLOV-Q489D with a minimum electron-electron distance of 9 ± 1 Å at low temperatures (Fig. 5). Room-temperature transient absorption (TA) spectroscopy of iLOV-Q489D identified this protein:FMN radical pair to be [FMNH^● …^ Trp^●^], suggesting the only Trp of iLOV-Q489D, W467, to be the electron donor for flavin photoreduction (Fig. 4). FMNH^●^ formation thereby occurs with the same kinetics like the decay of the triplet state, FMN^●−^ was not observed. Since hydrogen transfer is a rather unlikely process, we assume that the reaction in iLOV-Q489D proceeds as a sequence of electron transfer followed by proton transfer. The obvious conclusion then is that pT must be much faster than eT. We estimate that we should be able to see the radical anion intermediate in TA if it has a peak concentration of 10% of the concentration of the neutral radical. This corresponds to a ratio of the rate constants for formation and decay of the radical anion of *ca*. 0.14 or a lifetime of the radical anion of less than 3.8 µs. Electron transfer from a Trp residue to FMN and subsequent protonation via an aspartic acid nearby the FMN-N5 is known from plant cryptochromes (CRY)^56,57^. There, eT occurs via a Trp cascade in the ps time-range, while the protonation step varies between ns and µs^58,59^. In the case of the PHR domain of the algal CRY *Chlamydomonas* photolyase homologue 1, a lifetime of protonation of ca. 1.7 µs has been reported^58^, which was considered exceptionally slow. In other cases, however, very long lifetimes of the FMN radical anion have been observed where no proton is available for proton transfer^60^. Hence, we conclude that the proton provided by D489 neutralizes FMN^●−^ in iLOV-Q489D much faster than it is formed by eT.

In *A. thaliana* CRY the Trp triad is constituted by W400, W377 and W324^26,59^ located at an edge-to-edge distance of 4.5 Å, 9.7 Å and 14.7 Å from the FAD chromophore (see Supplementary Materials, Table S2), with the closest Trp (W400) representing the initial electron donor for FAD photoreduction^59^. In iLOV-Q489D, all redox-active amino acids, namely Y416, Y459, Y484 and W467, are located at an edge-to-edge distance of about 10 Å (see Supplementary Materials, Table S1). Likewise, photoreceptors of the BLUF family were suggested to undergo related photochemical reactions. While still under debate^3^, the BLUF photocycle may involve the ultrafast (ps) formation of FMNH^●^ as part of a radical-pair intermediate between the flavin and a conserved Tyr^61^. In iLOV-Q489D, the larger distance of W467 (10.8 Å) compared to W400 in AtCRY (4.5 Å) or Y8 of the Slr1694 BLUF protein (4.9 Å), could account for the eT reaction occurring in the µs-time range via the triplet state of FMN, compared to eT on the ps time scale from the singlet state in CRY^58,59^.

**Figure 7:**
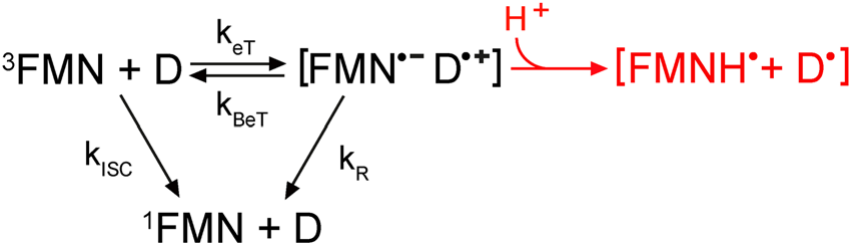
Proposed triplet-state mechanism of parental iLOV (black) and iLOV-Q489D (red). D represents the electron donor, i.e. Trp467. k_eT_: rate of eT; k_BeT_: rate of back electron transfer; k_R_: recombination rate; k_ISC_: rate of intersystem crossing.

In the present study, exchanging Q489 with Asp in the iLOV protein resulted in efficient FMN photoreduction yielding very stable FMNH^●^. Parental iLOV, on the other hand, did not possess any propensity for photoreduction in the presence of oxygen without the addition of the external electron donor EDTA in our steady-state UV/Vis experiments (Figure S2). In contrast to this, EPR spectroscopy at low temperatures reveals flavin radical formation in parental iLOV, although with a much lower amplitude compared to iLOV-Q489D (Fig. 5A). Also in our TA data, we could not detect any radical intermediate or transient species other than the FMN triplet state in the case of parental iLOV, but the triplet decay time is in the same time-range than the one of iLOV-Q489D (Fig. 3), suggesting that either the triplet state of parental iLOV has a quenching channel not present in iLOV-Q489D, or parental iLOV also undergoes eT. Partial quenching of the FMN triplet state has been observed in other LOV proteins lacking the reactive Cys^15,30,62^. There, varying accessibility of molecular oxygen into the binding pocket of the proteins has been suggested to constitute an additional deactivation channel of the FMN triplet state. We can rule out different oxygen accessibility in the case of iLOV since the dark reversion from FMNH^●^ to FMN_ox_ is very similar for parental iLOV and iLOV-Q489D (Figure S2D). Assuming eT occurring in parental iLOV, the radical anion formed by eT from the triplet state must decay to the ground state by a recombination process with the rate k_R_ that is faster than the rate of eT, k_eT_. As depicted in Fig. 7 (black text) one possible explanation is that ^3^FMN abstracts an electron from W467 forming the radical pair [FMN^●**−**^ Trp^●+^]. If k_eT_ ≫ k_BeT_ (the rate of back electron transfer) and k_eT_«k_R_, electron transfer represents the rate limiting step. The recombination of the radicals then is very fast and [FMN^●−^] ≪ [^3^FMN]. In this case, [FMN^●**−**^ Trp^●+^] could not be observed in our TA data. It should be noted that another possibility is k_BeT_ ≫ k_eT_. In this case, k_ISC_ (the rate of intersystem crossing) is the rate-limiting step. According to the ratio of the amplitudes of the two DADS of parental iLOV, more than 90% of the population of the ^3^FMN should be transferred to [FMN^●**−**^ Trp^●+^] in this case and should be visible by TA. Hence, we consider the first option to be more likely. The hypothesis of eT occurring in parental iLOV is supported by several solid-state and solution photo-CIDNP studies showing the formation of [FMN^●**−**^ Trp^●+^] in cysteine-less LOV domains of *C. reinhardtii* and *A. thaliana* ^52–55^. There, the electron donor could be identified to be the only Trp of the LOV domains with a comparable distance between FMN and Trp like in iLOV. According to these studies, it was not possible to detect the radical pair with other techniques like EPR or optical spectroscopy either due to the short lifetime of this radical pair or because the electron spin-relaxation is too fast for direct excitation.

In iLOV-Q489D however, k_BeT_ and k_R_ are suppressed due to the fast protonation of FMN-N5 by D489 (Fig. 7, red text), which removes the coulombic driving force for recombination. The difference in triplet decay times between iLOV-Q489D and parental iLOV could be due to the exchange of Gln with Asp: Replacing an amino side chain with an acidic side chain near FMN-N5 leads to an increase of the redox potential of the cofactor^63^. Hence, the driving force for electron transfer is larger in parental iLOV compared to iLOV-Q489D resulting in the faster triplet decay time.

Our mutational analysis reveals that substituting the redox-active amino acids in iLOV-Q489D, each at a time, has minor influence on the eT efficacy: the kinetics of FMNH^●^ formation in the steady-state UV/Vis experiments exhibit some variation when substituting Trp467 or one of the Tyr residues by Phe (Table 1). This can be due to conformational changes in the proteins induced by the mutations, influencing the distances and orientation of donor and acceptor for eT. For instance, it has been shown in the DASH-type cryptochrome CRYD that even subtle changes in the geometry or local sequence of amino acids can result in a diversity of eT pathways^64^.

Another possibility is that more than one of the mutated residues contributes to the photoreduction process. Hereby, Y416, Y459 and W467 could form a triad, similar to the Trp triad in CRY^26,59^. Our TA data, however, show that the counter radical of the eT to FMN is a Trp, except when the only Trp in iLOV is replaced by Phe, in which case the counter radical is Tyr. Furthermore, the mutation W467F significantly reduces the photoreduction yield (Table 1) and has strong impact on the lifetime of the FMN triplet state, which is not expected if eT from Tyr is the initial step in both variants. Although we cannot completely rule out the existence of an eT cascade in iLOV-Q489D, we prefer the interpretation that eT from Trp is more efficient than eT from Tyr, since the side chain of Trp has a greater reactivity towards the azide radical than the Tyr side chain. Hence, if both electron donors are present, indole oxidation is predominant^65^. As a result, in iLOV-Q489D eT from Tyr is only observed when Trp is no longer present. The decrease in driving force when Tyr is acting as the electron donor instead of Trp also explains the increase of the triplet decay time in iLOV-Q489D/W467F, where only Tyr are present. The distance between these Tyr and FMN is comparable to the one between W467 and FMN (see Supplementary Materials, Table S1). The electron transfer process in iLOV-Q489D/W467F becomes less efficient compared to iLOV-Q489D, but due to the fast protonation of FMN^●**−**^, FMNH^●^ is stabilized and can be detected via TA.

**Figure 8:**
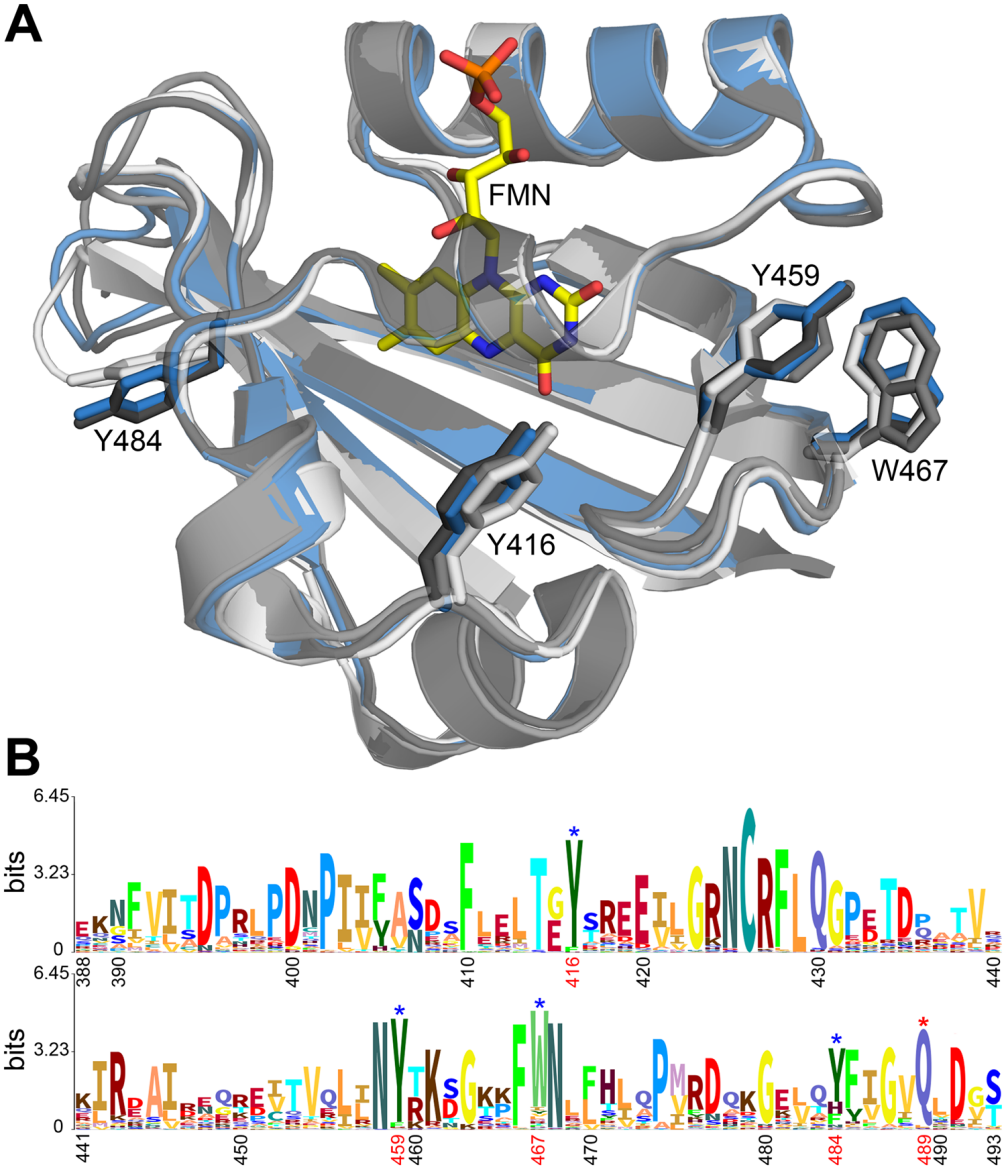
Superposition of representative LOV domain structures (**A**) and sequence logo generated to visualize the sequence conservation within the LOV domain family (**B**). The structure of the parent iLOV protein is shown as blue cartoon (PDB: 4EES). Additional LOV domain structures are shown in different shades of grey (from light to dark grey: LOV domain of YF1 (PDB: 4GCZ), *Chlamydomonas rheinhardtii* phototropin LOV1 (PDB: 1N9L), LOV2 domain of *Avena sativa* phototropin 2 (PDB: 2V0U). The residues corresponding to Y416, Y459, Y484 and W467 of parental iLOV are shown in stick representation in the corresponding color. The sequence logo shown in panel (**B**) was generated using the Skylign tool^70^ using a multiple sequence alignment containing 1975 LOV domain sequences taken from^7^. Sequence positions are numbered according to iLOV numbering. Amino acids relevant for the discussion are marked by blue (potential electron donating residues) and red (conserved glutamine Q489) asterisks and numbered in red.

Recently, a comprehensive LOV protein sequence dataset was provided by Glantz and co-workers^7^. To address the issue of conservation of the respective eT and pT reactions observed in iLOV-Q489D, we analyzed this dataset for conservation of the respective amino acid positions. Figure 8 depicts the sequence logo obtained from a multiple sequence alignment of 1975 LOV domain sequences extracted from^7^ as well as a superposition of various LOV domain structures highlighting the structural conservation of the respective residues.

The conservation analysis suggests that the residues constituting the eT pathway in iLOV-Q489D are highly conserved among LOV domains (Fig. 8, B marked by blue asterisk). Likewise, Q489 is highly conserved (Fig. 8, B, red asterisk), i.e. no potential proton donating residues are found at the respective positions, suggesting that evolution eventually has favored a Cys-adduct dependent LOV photochemistry over FMN photoreduction.

In the present study, efficient FMN photoreduction could readily be engineered into the iLOV protein, which in its “native” form did not possess any propensity for photoreduction in the presence of oxygen. Hereby, introduction of a potentially proton donating Asp residue close to the FMN chromophore appears to be of primary importance as the small size of the LOV domain apparently provides ample possibilities for eT from redox-active amino acids. Sequence analyses suggested that the eT pathway involving multiple Tyr and Trp residues is conserved among LOV proteins. Furthermore, our findings suggest that, while the propensity for photoreduction is evolutionary imprinted in all LOV domains, the efficacy of FMNH^●^ formation, which varies widely in the corresponding cysteine to alanine mutants of different LOV domains, is determined by the possibility for an efficient pT either from an amino acid residue in the proximity of FMN chromophore (as in iLOV-Q489D) or from a water molecule to stabilize FMNH^●^. In the latter case, a subtle balance would be needed between rigid anchoring of the FMN chromophore (for efficient eT) and the access of water to enable pT. The apparent easy adaptability of the LOV photochemistry to follow a route with intriguing similarity to other flavin-binding photoreceptors such as CRY and BLUF provides a rational basis for the evolutionary success of the flavin cofactor to act as light absorbing chromophore in various structurally and evolutionary unrelated blue-light photoreceptors, and underpins the hypothesis that todays’ adduct forming LOV photoreceptors evolutionary derive from ancestral redox-active flavoproteins^17^.

## Materials and Methods

### Bacterial strains, plasmids and site-directed mutagenesis

All bacterial strains used in this study were either grown in Luria-Bertani (LB) broth or in autoinduction (AI) terrific broth (TB) media as described previously^29,31^. The gene coding for the parent iLOV protein (Addgene plasmid pGEX-iLOV (Addgene ID: 26587) and the iLOV-Q489D variant encoding gene were obtained in custom synthesized form from Life Technologies (Thermo Fisher Scientific Inc, Waltham, MA, USA). Both genes were flanked by a 5′- *Nde*I and a 3′- *Sal*I restriction endonuclease recognition site. The Q489D variant was generated by including an aspartate codon (nucleotide sequence: GAT) instead of the parental Q489 codon (nucleotide sequence: CAG) during gene synthesis. The codon-usage was not optimized during gene synthesis. The restriction endonucleases *Nde*I and *Sal*I were utilized to subclone the respective synthetic genes into pET28a as expression vector (Novagen/Merck, Darmstadt, Germany) resulting in pET28_iLOV and pET28a_iLOV-Q489D, respectively. The double variants iLOV-Q489D/Y416F, iLOV-Q489D/Y459F, iLOV-Q489D/Y484F and iLOV-Q489D/W467F were generated by Quikchange PCR according to the instructions given by the manufacturer (Stratagene, La Jolla, CA). Oligonucleotide sequences as well as detailed procedures are described in the Supporting Information. The corresponding gene products contained a 20 amino acid N-terminal hexa-histidine tag (His6), which is encoded on the pET28a plasmid. All final expression vectors were verified by sequencing (SeqLab GmbH, Göttingen, Germany).

### Protein production and purification

All constructs were expressed as His_6_-tagged fusion proteins in *E. coli* BL21(DE3) to facilitate purification by immobilized metal-ion affinity chromatography (IMAC) as described previously^29,31^. Purified protein samples were stored at 4 °C in 10 mM sodium phosphate buffer, pH 8.0 supplemented with 10 mM NaCl (storage buffer) or 200 mM sodium phosphate buffer, pH 7.2 supplemented with 10 mM NaCl.

### Photoreduction of parental iLOV and iLOV-Q489D under aerobic and anaerobic conditions

Steady-state UV/Vis spectrophotometric measurements were carried out under dim-red safety light using a Cary-60 UV/Vis spectrophotometer (Agilent Technologies, Santa Clara, CA, USA) equipped with a peltier-thermostatted (25 ± 2 °C) single-cell cuvette holder. All samples were kept in the dark for at least 2 hours before use. All iLOV-Q489D samples were diluted (1:10) to an OD_450nm_ of 0.1 in 200 mM sodium phosphate buffer pH 7.2 supplemented with 10 mM NaCl. For the measurement under aerobic conditions sample illumination was achieved by using a blue-light emitting high-power LED (Luxeon Lumileds, Phillips, Aachen, Germany) (0.13 mW cm^−2^) mounted on top of the cuvette. LED illumination was controlled using an Arduino UNO (Smart Projects, Italy) microcontroller as described previously^66^. Illumination was pulsed (5 seconds illumination time) to precede each sequential UV/Vis scan and spectra were recorded every 30 seconds for up to 15 minutes. To monitor photoreduction in the absence of oxygen, the respective diluted protein solution was degassed by bubbling the solution for 15 minutes with Argon in a 1 cm quartz cuvette closed with a rubber septum. The corresponding degassed protein solution was illuminated for up to 45 minutes by removing the cuvette from the sample holder and illuminating the solution from the side of the cuvette.

### pH-dependent photoreduction under aerobic conditions

All UV/Vis spectrophotometric measurements were carried as described above. All iLOV-Q489D samples were diluted (1:10) to an OD_450nm_ of 0.1 using buffers of the appropriate pH. For measurements in the range of pH 2.6–7.0, samples were diluted with 200 mM citrate/phosphate buffer supplemented with 10 mM NaCl. For measurements at pH 8.0, 200 mM sodium phosphate buffer supplemented with 10 mM NaCl was employed for dilution. In the pH range from 8.6 to 10.6, samples were diluted with 200 mM glycine/NaOH buffer supplemented with 10 mM NaCl. The final pH for each measurement was verified using mock samples containing storage buffer instead of protein using an identical dilution as employed for sample measurements. Sample illumination was achieved as described above. To account for pH-dependent unfolding, samples were diluted as described above and sequential UV/Vis spectra were recorded.

### Transient absorption (TA) spectroscopy

Transient absorption spectroscopy (TA) in the µs-time range was performed using a streak camera (C7700, Hamamatsu Photonics) setup. The samples were excited at 447 nm using an OPO pumped by a Nd:YAG laser (Surelite II, Continuum) with a pulse width of 8 ns. The laser energy was adjusted to be around 10 mJ/pulse in front of the cuvette. The protein solutions were pumped through a fused silica cuvette with 2 mm optical length for excitation and 10 mm path length for probe light during the measurements using a peristaltic pump in order to exchange the sample volume between two excitation cycles. Samples were diluted to an optical density of ~0.5 at 450 nm in a total volume of ca. 10 mL. Each measurement was performed as a sequence of 100 excitation cycles with one cycle consisting of four individual streak images taken with a frequency of 1 Hz: *I*_*L*_,_*P*_, *I*_*D*_, *I*_*P*_, *I*_*D*_. *I*_*L*_,_*P*_ refers to an image with both laser and probe light, *I*_*D*_ being a dark spectrum and *I*_*P*_ probe light only. iLOV data were collected on a 200 µs time window. The setup is described in further detail in^67^.

For the Trp^●^/Cys^●^ reference spectrum, FMN was dissolved in 10 mM sodium phosphate buffer, pH 8.0 supplemented with 10 mM NaCl at a concentration of 40 µM, resulting in an optical density of 0.5 at 450 nm. To this solution either L-Trp or L-Cys was added at a concentration of 100 mM. TA was measured on a 20 µs time window.

### Electron paramagnetic resonance (EPR) spectroscopy

EPR measurements were performed with a Bruker ELEXSYS E580 X-band EPR spectrometer equipped with the Oxford helium flow cryostat and the dielectric EN 4118X-MD5 EPR resonator at 80 K. The sample solutions in quartz tubes (4.8 mm O.D. and 3.8 mm I.D.) were inserted into the precooled EPR resonator. Sample illumination was performed using the output of InnoLas laser equipped with OPO at 450 nm, 10 Hz, 5 mJ on the sample surface. Continuous wave (cw) EPR spectra were recorded at 120 K using low microwave power of 4 µW in order to avoid saturation and 0.3 mT magnetic field modulation at 100 kHz. All pulsed EPR experiments were performed at 60 K. Echo-detected (ED) field-swept spectra were acquired at 120 K with the Hahn-echo pulse sequence *t*_*p*_−τ−2*t*_*p*_−*τ*−echo using *t*_*p*_ = 20 ns and *τ* = 150 ns. Electron spin nutation experiments were performed with the *t*_*prep*_−*Τ*−*t*_*p*_−*τ*−2*t*_*p*_−*τ*−echo sequence using *t*_*prep*_ = 2–1400 ns incremented in 2 ns steps, *t*_*p*_ = 30 ns, *T* = 3 ms and *τ* = 800 ns.

### Data analysis

#### Determination of photoreduction yield and FMNH^●^ formation kinetics

Photoreduction yields at a given pH, quantifying the conversion of oxidized FMN (FMN_ox_) to the neutral FMN semiquinone radical (FMNH^●^), were obtained from UV/Vis spectra by determining the rise in absorbance at 615 nm due to illumination over 150 s illumination time. Spectra were normalized to the initial absorbance of the oxidized flavin at 450 nm, to account for slight differences in protein concentration due to dilution. The photoreduction lifetime for FMNH^●^ formation τ_Sq_ at a given pH was obtained by plotting the rise in absorbance at 615 nm (Abs_615nm_) against the illumination time and fitting the data to a single exponential decay function employing Origin 9.0 G (OriginLab corporation, Northampton, MA, USA) using the following equation:

3 $${{\rm{Abs}}}_{615{\rm{nm}}}({\rm{t}})={{\rm{Abs}}}_{615{\rm{nm}}}({\rm{t}}=0)+{{\rm{A}}}_{1}\times {{\rm{e}}}^{(\frac{{\rm{t}}}{{{\rm{\tau }}}_{{\rm{Sq}}}})}\,$$

#### Analysis of TA data

The two dimensional data sets obtained from TA measurements were analyzed using home-written software. The details are described in ref.^67^ In short, global lifetime analysis is applied to the data to obtain the decay associated difference spectra (DADS) by fitting the data to the expression:

4 $${\rm{\Delta }}A(t,\lambda )=({D}_{0}(\lambda )\,\delta (t)+{\sum }_{j=1}^{N}{D}_{j}\,(\lambda )\,\exp \,(-{k}_{j}t))\otimes {g}_{app}\,(t-{t}_{0})$$

where *ΔA*(*t,λ*) refers to the measured data matrix, $\otimes {g}_{app}(t-{t}_{0})$ indicates convolution with the apparatus function approximated by a Gaussian, $\delta (t)$ is the Dirac delta function and *N* is the number of decay components considered. The result of this fit are the rate constants *k*_*j*_ = *1*/*τ*_*j*_ and the corresponding decay associated difference spectra (DADS), *D*_*j*_(*λ*). The spectrum *D*_0_(*λ*) accounts for the contribution of processes that are faster than the time resolution of the apparatus, e.g. fluorescence and laser stray light. The resolution of the streak camera of 1 pixel corresponds to a time of ca. 400 ns for the time windows chosen. A least-square algorithm was used in order to identify the species that contribute to DADS2 of iLOV-Q489D (Fig. 3). A linear combination of reference spectra was formed to calculate the model spectrum, and the coefficients were optimized. The reference spectra for FMN_ox_ and FMNH^●^ were taken from the steady state measurements. The signal to noise ratio of the steady state UV/Vis spectra is much better than the reference spectrum of Trp^●^ obtained by TA. Therefore, the noise level of the Trp^●^ was reduced by singular value decomposition (SVD) prior to fitting.

#### Analysis of conservation of amino acid residues

To construct a comprehensive multiple sequence alignment for LOV domain sequences, we made use of the recently published LOV amino acid sequence dataset presented by Glantz *et al*.^7^ The second Excel table in the supplement of^7^ contains 5499 non-redundant LOV photoreceptor sequences, from which we extracted the LOV/TLOV domain according to the domain annotation given in the table. Reduction of redundancy below 94% with Jalview^68^ reduced the number of sequences to 1983 sequences. A multiple sequence alignment was generated using MAFFT-FFT-NS-I^69^ in Jalview. Additionally, some sequences with insertions around the catalytic Cys were manually deleted to remove gaps, resulting in an alignment of 1975 LOV domain sequences. The sequence logo was generated with Skylign^70^.

### Data Availability

The datasets generated during and/or analysed during the current study are available from the corresponding author on reasonable request.

## Electronic supplementary material

Supporting Information

## References

[1] Fraaije MW, Mattevi A (2000). Flavoenzymes: diverse catalysts with recurrent features. Trends in biochemical sciences.

[2] Massey V (2000). The chemical and biological versatility of riboflavin. Biochemical Society transactions.

[3] Conrad KS, Manahan CC, Crane BR (2014). Photochemistry of flavoprotein light sensors. Nature chemical biology.

[4] Henry JT, Crosson S (2011). Ligand-binding PAS domains in a genomic, cellular, and structural context. Annual review of microbiology.

[5] Losi A, Gärtner W (2011). Old chromophores, new photoactivation paradigms, trendy applications: flavins in blue light-sensing photoreceptors. Photochemistry and photobiology.

[6] Zoltowski BD, Gardner KH (2011). Tripping the Light Fantastic: Blue-Light Photoreceptors as Examples of Environmentally Modulated Protein-Protein Interactions. Biochemistry-Us.

[7] Glantz, S. T. *et al*. Functional and topological diversity of LOV domain photoreceptors. *Proceedings of the National Academy of Sciences of the United States of America*, 10.1073/pnas.1509428113 (2016). 10.1073/pnas.1509428113 PMC4801262 26929367

[8] Krauss U (2009). Distribution and phylogeny of light-oxygen-voltage-blue-light-signaling proteins in the three kingdoms of life. Journal of bacteriology.

[9] Möglich A, Yang X, Ayers RA, Moffat K (2010). Structure and function of plant photoreceptors. Annual review of plant biology.

[10] Endres S (2015). Structure and function of a short LOV protein from the marine phototrophic bacterium Dinoroseobacter shibae. BMC microbiology.

[11] Herrou J, Crosson S (2011). Function, structure and mechanism of bacterial photosensory LOV proteins. Nature reviews. Microbiology.

[12] Rivera-Cancel G, Ko WH, Tomchick DR, Correa F, Gardner KH (2014). Full-length structure of a monomeric histidine kinase reveals basis for sensory regulation. Proceedings of the National Academy of Sciences of the United States of America.

[13] Swartz TE (2001). The photocycle of a flavin-binding domain of the blue light photoreceptor phototropin. The Journal of biological chemistry.

[14] Bauer C, Rabl CR, Heberle J, Kottke T (2011). Indication for a Radical Intermediate Preceding the Signaling State in the LOV Domain Photocycle. Photochemistry and photobiology.

[15] Kutta, R. J., Magerl, K., Kensy, U. & Dick, B. A search for radical intermediates in the photocycle of LOV domains. *Photochemical & photobiological sciences* **14**, 288–299, 10.1039/c4pp00155a (2015). 10.1039/c4pp00155a 25380177

[16] Chang, X. P., Gao, Y. J., Fang, W. H., Cui, G. & Thiel, W. Quantum Mechanics/Molecular Mechanics Study on the Photoreactions of Dark- and Light-Adapted States of a Blue-Light YtvA LOV Photoreceptor. *Angewandte Chemie*, 10.1002/anie.201703487 (2017). 10.1002/anie.201703487 28632317

[17] Yee EF (2015). Signal transduction in light-oxygen-voltage receptors lacking the adduct-forming cysteine residue. Nature communications.

[18] Cho HY (2007). Physiological roles of the light, oxygen, or voltage domains of phototropin 1 and phototropin 2 in Arabidopsis. Plant physiology.

[19] Okajima K (2014). Light-induced conformational changes of LOV1 (light oxygen voltage-sensing domain 1) and LOV2 relative to the kinase domain and regulation of kinase activity in Chlamydomonas phototropin. The Journal of biological chemistry.

[20] Aihara Y (2012). Mutations in N-terminal flanking region of blue light-sensing light-oxygen and voltage 2 (LOV2) domain disrupt its repressive activity on kinase domain in the Chlamydomonas phototropin. The Journal of biological chemistry.

[21] Song SH, Dick B, Penzkofer A, Hegemann P (2007). Photo-reduction of flavin mononucleotide to semiquinone form in LOV domain mutants of blue-light receptor phot from Chlamydomonas reinhardtii. Journal of photochemistry and photobiology. B, Biology.

[22] Kay CW (2003). Blue light perception in plants. Detection and characterization of a light-induced neutral flavin radical in a C450A mutant of phototropin. The Journal of biological chemistry.

[23] Lanzl K, Sanden-Flohe MV, Kutta RJ, Dick B (2010). Photoreaction of mutated LOV photoreceptor domains from Chlamydomonas reinhardtii with aliphatic mercaptans: implications for the mechanism of wild type LOV. Physical chemistry chemical physics: PCCP.

[24] Noll G (2007). Redox properties of LOV domains: chemical versus photochemical reduction, and influence on the photocycle. Chembiochem: a European journal of chemical biology.

[25] Chapman S (2008). The photoreversible fluorescent protein iLOV outperforms GFP as a reporter of plant virus infection. Proceedings of the National Academy of Sciences of the United States of America.

[26] Chaves I (2011). The cryptochromes: blue light photoreceptors in plants and animals. Annual review of plant biology.

[27] Drepper T (2007). Reporter proteins for *in vivo* fluorescence without oxygen. Nat Biotechnol.

[28] Christie JM (2012). Structural tuning of the fluorescent protein iLOV for improved photostability. The Journal of biological chemistry.

[29] Wingen M (2014). The photophysics of LOV-based fluorescent proteins–new tools for cell biology. Photochemical & photobiological sciences: Official journal of the European Photochemistry Association and the European Society for Photobiology.

[30] Magerl K, Stambolic I, Dick B (2017). Switching from adduct formation to electron transfer in a light-oxygen-voltage domain containing the reactive cysteine. Physical Chemistry Chemical Physics.

[31] Davari MD (2016). Photophysics of the LOV-Based Fluorescent Protein Variant iLOV-Q489K Determined by Simulation and Experiment. The journal of physical chemistry. B.

[32] Iqbal A, Gomes-Neto F, Myiamoto CA, Valente AP, Almeida FC (2015). Dissection of the water cavity of yeast thioredoxin 1: the effect of a hydrophobic residue in the cavity. Biochemistry-Us.

[33] Jeng M-F, Dyson HJ (1996). Direct Measurement of the Aspartic Acid 26 pKa for Reduced Escherichia coli Thioredoxin by 13C NMR. Biochemistry-Us.

[34] Chivers PT (1997). Microscopic pKa values of Escherichia coli thioredoxin. Biochemistry-Us.

[35] Olsson MH, Søndergaard CR, Rostkowski M, Jensen JH (2011). PROPKA3: consistent treatment of internal and surface residues in empirical pKa predictions. Journal of Chemical Theory and Computation.

[36] Sakai M, Takahashi H (1996). One-electron photoreduction of flavin mononucleotide: time-resolved resonance Raman and absorption study. Journal of Molecular Structure.

[37] Liu Z, Wang L, Zhong D (2015). Dynamics and mechanisms of DNA repair by photolyase. Physical Chemistry Chemical Physics.

[38] Müller, P., Brettel, K., Grama, L., Nyitrai, M. & Lukacs, A. Photochemistry of Wild-Type and N378D Mutant E. coli DNA Photolyase with Oxidized FAD Cofactor Studied by Transient Absorption Spectroscopy. *ChemPhysChem*, n/a-n/a, 10.1002/cphc.201501077 (2016). 10.1002/cphc.201501077 26852903

[39] Aubert C, Mathis P, Eker APM, Brettel K (1999). Intraprotein electron transfer between tyrosine and tryptophan in DNA photolyase from Anacystis nidulans. Proceedings of the National Academy of Sciences.

[40] Bent DV, Hayon E (1975). Excited state chemistry of aromatic amino acids and related peptides. I. Tyrosine. Journal of the American Chemical Society.

[41] Bernt Melø T, Adriana Ionescu M, Haggquist GW, Razi Naqvi K (1999). Hydrogen abstraction by triplet flavins. I: time-resolved multi-channel absorption spectra of flash-irradiated riboflavin solutions in water. Spectrochimica Acta Part A: Molecular and Biomolecular Spectroscopy.

[42] Solar S, Getoff N, Surdhar PS, Armstrong DA, Singh A (1991). Oxidation of tryptophan and N-methylindole by N3.cntdot., Br2.-, and (SCN)2.- radicals in light- and heavy-water solutions: a pulse radiolysis study. The Journal of Physical Chemistry.

[43] Liu X (2014). Significant expansion of fluorescent protein sensing ability through the genetic incorporation of superior photo-induced electron-transfer quenchers. J Am Chem Soc.

[44] Kay CW (1999). EPR, ENDOR, and TRIPLE resonance spectroscopy on the neutral flavin radical in Escherichia coli DNA photolyase. Biochemistry-Us.

[45] Bleifuss G (2001). Tryptophan and tyrosine radicals in ribonucleotide reductase: A comparative high-field EPR study at 94 GHz. Biochemistry-Us.

[46] Pogni R (2006). A tryptophan neutral radical in the oxidized state of versatile peroxidase from Pleurotus eryngii: a combined multifrequency EPR and density functional theory study. The Journal of biological chemistry.

[47] Pogni R (2005). Tryptophan-based radical in the catalytic mechanism of versatile peroxidase from Bjerkandera adusta. Biochemistry-Us.

[48] Lendzian F (1996). Electronic structure of neutral tryptophan radicals in ribonucleotide reductase studied by EPR and ENDOR spectroscopy. Journal of the American Chemical Society.

[49] Schweiger, A. & Jeschke, G. *Principles of Pulse Electron Paramagnetic Resonance* (Oxford Univ. Press, Oxford, UK, 2001).

[50] Takui TS, Shiomi K, Itoh D, Kaneko K, Tsuchida T, Nishide E (1996). H. FT Pulsed ESR/Electron Spin Transient Nutation (ESTN) Spectroscopy Applied to High-Spin Systems in Solids; Direct Evidence of a Topologically Controlled High-Spin Polymer as Models for Quasi ID Organic Ferro- and Superparamagnets. Molecular Crystals and Liquid Crystals Science and Technology. Section A: Molecular Crystals and Liquid Crystals.

[51] Proshlyakov DA (2004). UV optical absorption by protein radicals in cytochrome c oxidase. Biochimica et Biophysica Acta (BBA) - Bioenergetics.

[52] Eisenreich W (2009). Tryptophan 13C nuclear-spin polarization generated by intraprotein electron transfer in a LOV2 domain of the blue-light receptor phototropin. Biochemical Society transactions.

[53] Eisenreich W, Joshi M, Weber S, Bacher A, Fischer M (2008). Natural Abundance Solution 13C NMR Studies of a Phototropin with Photoinduced Polarization. Journal of the American Chemical Society.

[54] Richter G (2005). Photochemically Induced Dynamic Nuclear Polarization in a C450A Mutant of the LOV2 Domain of the Avena sativa Blue-Light Receptor Phototropin. Journal of the American Chemical Society.

[55] Thamarath SS, Heberle J, Hore PJ, Kottke T, Matysik J (2010). Solid-State Photo-CIDNP Effect Observed in Phototropin LOV1-C57S by 13C Magic-Angle Spinning NMR Spectroscopy. Journal of the American Chemical Society.

[56] Immeln D, Weigel A, Kottke T, Pérez Lustres JL (2012). Primary Events in the Blue Light Sensor Plant Cryptochrome: Intraprotein Electron and Proton Transfer Revealed by Femtosecond Spectroscopy. Journal of the American Chemical Society.

[57] Maeda K (2012). Magnetically sensitive light-induced reactions in cryptochrome are consistent with its proposed role as a magnetoreceptor. Proceedings of the National Academy of Sciences.

[58] Langenbacher T, Immeln D, Dick B, Kottke T (2009). Microsecond Light-Induced Proton Transfer to Flavin in the Blue Light Sensor Plant Cryptochrome. Journal of the American Chemical Society.

[59] Solov’yov IA, Domratcheva T, Moughal Shahi AR, Schulten K (2012). Decrypting Cryptochrome: Revealing the Molecular Identity of the Photoactivation Reaction. Journal of the American Chemical Society.

[60] Hense A, Herman E, Oldemeyer S, Kottke T (2015). Proton Transfer to Flavin Stabilizes the Signaling State of the Blue Light Receptor Plant Cryptochrome. Journal of Biological Chemistry.

[61] Gauden M (2006). Hydrogen-bond switching through a radical pair mechanism in a flavin-binding photoreceptor. Proceedings of the National Academy of Sciences of the United States of America.

[62] Torra J (2015). *Singlet oxygen photosensitisation by the fl*uorescent protein Pp2FbFP L30M, a novel derivative of Pseudomonas putida flavin-binding Pp2FbFP. Photochemical & Photobiological Sciences.

[63] Balland V, Byrdin M, Eker APM, Ahmad M, Brettel K (2009). What Makes the Difference between a Cryptochrome and DNA Photolyase? A Spectroelectrochemical Comparison of the Flavin Redox Transitions. Journal of the American Chemical Society.

[64] Biskup T (2011). Unexpected Electron Transfer in Cryptochrome Identified by Time-Resolved EPR Spectroscopy. Angewandte Chemie International Edition.

[65] Prütz WA, Land EJ (1979). Charge Transfer in Peptides. International Journal of Radiation Biology and Related Studies in Physics, Chemistry and Medicine.

[66] Kaschner M (2014). Discovery of the first light-dependent protochlorophyllide oxidoreductase in anoxygenic phototrophic bacteria. Molecular microbiology.

[67] Kutta R-J, Langenbacher T, Kensy U, Dick B (2013). Setup and performance of a streak camera apparatus for transient absorption measurements in the ns to ms range. Applied Physics B.

[68] Waterhouse AM, Procter JB, Martin DM, Clamp M, Barton GJ (2009). Jalview Version 2–a multiple sequence alignment editor and analysis workbench. Bioinformatics.

[69] Katoh K, Misawa K, Kuma KI, Miyata T (2002). MAFFT: a novel method for rapid multiple sequence alignment based on fast Fourier transform. Nucleic acids research.

[70] Wheeler, T. J., Clements, J. & Finn, R. D. Skylign: a tool for creating informative, interactive logos representing sequence alignments and profile hidden Markov models. *BMC bioinformatics* **15**, 1, 10.1186/1471-2105-15-7 (2014). 10.1186/1471-2105-15-7 PMC3893531 24410852

