# Supplementary material for: Electron transfer pathways in a light, oxygen, voltage (LOV) protein devoid of the photoactive cysteine
Source: Sci Rep. 2017 Oct 17;7:13346. doi: 10.1038/s41598-017-13420-1 (PMC5645311; doi:10.1038/s41598-017-13420-1)
Supplement: Supplementary file 1 — Supporting Information [file 41598_2017_13420_MOESM1_ESM.pdf]

Supporting Information

Electron transfer pathways in a light, oxygen, voltage (LOV) protein devoid of the photoactive cysteine

Benita Kopka[1#], Kathrin Magerl[2#], Anton Savitsky[3], Mehdi D. Davari[4], Katrin Röllen[1], Marco Bocola[4], Bernhard Dick[2], Ulrich Schwaneberg[4,5], Karl-Erich Jaeger[1,6] and Ulrich Krauss[1*]

* Corresponding author

[#] contributed equally

[1]Institut für Molekulare Enzymtechnologie, Heinrich-Heine-Universität Düsseldorf, Forschungszentrum Jülich, 52426 Jülich, Germany

[2]Institut für Physikalische und Theoretische Chemie, Universität Regensburg, 93053 Regensburg, Germany

[3]Max Planck Institute for Chemical Energy Conversion, 45470 Mülheim an der Ruhr, Germany

[4]Lehrstuhl für Biotechnologie, RWTH Aachen University, Worringerweg 3, 52074 Aachen, Germany

[5]DWI-Leibniz Institute for Interactive Materials, Forckenbeckstraße 50, 52056, Aachen, Germany

[6]IBG-1: Biotechnologie, Forschungszentrum Jülich, 52426 Jülich, Germany

content	description	page
	**Supporting Methods**	
**Supporting Methods**	Site directed mutagenesis<br>Determination of fluorescence lifetimes using time correlated single photon counting (TCSPC)<br>Analysis of TCSPC data<br>Ultra-High-Performance Liquid Chromatography (UPLC) analyses.<br>Singular value decomposition (SVD) and deconvolution of time sequences of the steady-state UV/Vis spectra of iLOV-Q489D at pH 7.2<br>Molecular dynamics (MD) simulations	**S4-S8**
	**Supporting Data and Figures**	
**Figure S1**	Close-up view of the FMN binding pocket of iLOV	**S9**
**Figure S2**	Photoreduction of parental iLOV in the presence and absence of oxygen, Photoreduction of parental iLOV under anaerobic conditions in the presence of EDTA, Reoxidation kinetics of parental iLOV and iLOV-Q489D	**S10**
	**Supporting Results:** pH-dependence of iLOV-Q489D photoreduction	**S11-S13**
**Figure S3**	Spectral changes associated with illumination of iLOV-Q489D at pH 2.6 to pH 10.6.	**S12**
**Figure S4**	pH stability of iLOV-Q489D at pH 2.6, pH 3.0, pH 9.0 and pH 10.6.	**S13**
	**Supporting Results:** Spectral changes associated with photoreduction of iLOV-Q489D at pH 7.2, pH 2.8 and pH 9.8	**S14-S20**
**Figure S5**	Photoreduction of iLOV-Q489D and FMN reoxidation at pH 7.2, pH 2.8 and pH 9.8.	**S15**
**Figure S6**	SVD analysis of the photoreduction data of iLOV-Q489D at pH 7.2	**S16**
**Figure S7**	UPLC analysis of iLOV-Q489D at pH 7.2 and after photoreduction at pH 2.6 and 9.8.	**S17**
	**Supporting Results:** UV/Vis spectra along with computational predictions suggest that the newly introduced Asp is protonated at neutral pH values	**S18-S19**
**Figure S8**	UV/Vis spectra of parental iLOV, iLOV-Q489D at pH 7.2; Close-up view of the FMN binding pocket of iLOV-Q489D; H-bond analysis	**S19**
	**Supporting Results:** Generation of Trp$^{\bullet}$ reference spectrum in solution	**S20**
**Figure S9**	Comparison of the difference absorption spectra of FMN + Trp and FMN + Cys in the absence of apoprotein.	**S20**
	**Supporting Results:** Electron paramagnetic resonance (EPR) spectroscopy reveals the presence of a stable radical pair in iLOV-Q489D	**S21-22**
**Figure S10**	X-band echo detected EPR spectra of parental iLOV and iLOV-Q489D	**S21**
**Figure S11**	X-band continuous wave (cw) EPR spectra of iLOV-Q489D	**S22**
	**Supporting Results:** Experimental and computational analysis of the eT pathway in iLOV-Q489D	**S23-S28**
**Figure S12**	Potential electron donating Tyr and Trp residues in parental iLOV	**S23**
**Table S1**	Center of mass and edge-to-edge distances between the redox active amino acids Y416, Y459, Y484, W467 and the FMN chromophore in iLOV	**S24**
**Figure S13**	Photoreduction and reoxidation of iLOV-Q489D, iLOV-Q489D/Y416F, iLOV-Q489D/Y459F, iLOV-Q489D/Y484F and iLOV-Q489D/W467F.	**S25**
**Figure S14**	DADS of iLOV-Q489D/W467F obtained via global analysis of a 2D TA data set measured on a 200 µs streak window	**S26**
**Figure S15**	Close-up view of the crystal structure of *A. thaliana* Cry1 (PDB: 1U3D)	**S27**
**Table S2**	Center of mass (black) and edge-to-edge (red) distances between the components of the Trp triad in *Arabidopsis thaliana* Cry1	**S28**

**Supporting Results:** Molecular dynamic (MD) simulations of iLOV-Q489D		**S29-S32**
**Figure S16**	Interatomic distances between FMN-O4…D489-OD2 and FMN-N5…D489-OD2 calculated over the MD trajectories of iLOV-Q489D with protonated D489	**S29**
**Figure S17**	Interatomic distances between FMN-O4…D489-OD2 and FMN-N5…D489-OD2 calculated over the MD trajectories of iLOV-Q489D with deprotonated D489	**S30**
**Figure S18**	Distance distribution curve of the interatomic distances between FMN-O4…D489-OD2 and FMN-N5…D489-OD2 calculated over the MD trajectories of iLOV-Q489D with protonated D489	**S31**
**Figure S19**	Distance distribution curve of the interatomic distances between FMN-O4…D489-OD2 and FMN-N5…D489-OD2 calculated over the MD trajectories of iLOV-Q489D with deprotonated D489	**S32**
**Supporting References**		**S33**

Supplementary Methods

Site directed mutagenesis. The double variants iLOV-Q489D/Y416F, iLOV-Q489D/Y459F, iLOV-Q489D/Y484F and iLOV-Q489D/W467F were generated by Quikchange PCR according to the instructions given by the manufacturer (Stratagene, La Jolla, CA). The plasmid pET28a_iLOV-Q489D was used as template together with the primers QC_iLOV-Y416F_f (5'-GACAGAGTTTTCGCG CGAGGAAATATTGGG-3') and QC_iLOV-Y416F_r (5'-CGCGCGAAAACTCTGTCA ATTCAAGAAAGC-3'), QC_iLOV-Y459F_f (5'-GATAAACTTCACTAA AAG CGGAAA GAAATT-3') and QC_iLOV-Y459F_r (5'-TTTTAGTGAAGTTTATCAACTGCACAG TAG-3'), QC_iLOV-Y484F_f (5'-GCTTCAATTCTTCATCGGTGTGGATCTCGA-3') and QC_iLOV-Y484F_r (5'-CGATGAAGAATTGAAGCTCTCCCTTCTGAT -3'), QC_iLOV-W467F_f (5'-GAAATTCTTTAACTTACTCCACCTGCAACC-3') and QC_iLOV-W467F_r (5'-GTAAGTTAAAGAATTTCTTTCCGCTTTTAG-3') to generate iLOV-Q489D/Y416F, iLOV-Q489D/Y459F, iLOV-Q489D/Y484F and iLOV-Q489D/W467F, respectively. For PCR amplification Phusion high-fidelity DNA polymerase (ThermoFisher Scientific, Waltham, MA, USA) was employed. Parental template DNA was digested using the restriction endonuclease *Dpn*I. All final constructs were verified by sequencing (SeqLab GmbH, Göttingen, Germany).

Reoxidation under aerobic conditions. To evaluate the efficacy of FMN reoxidation after complete photoreduction, photoreduction measurements were carried out as described in the main manuscript. Subsequently the light was switched off and flavin reoxidation was monitored for 2 hours by recording a UV/Vis spectrum every 2 minutes.

The efficacy of the process was quantified by determining the reoxidition lifetime τ_{FMNox} by plotting the rise in absorbance at 450 nm after photoreduction due to reoxidation (Abs_{450nm}) against the time in the dark and fitting the experimental data to a single-exponential decay function:

$$Abs_{450nm}(t) = Abs_{450nm}(t=0) + A_1 \times e^{\left(\frac{t}{\tau_{FMNox}}\right)} \quad (1)$$

Determination of fluorescence lifetimes using time correlated single photon counting (TCSPC). The fluorescence lifetimes of the excited S_1 states were determined by TCSPC with a time resolution in the ns – range at room temperature. The samples were excited at 443 nm with a pulsed LED (NanoLED-450, Horiba Jobin Yvon, λ_{max} = 443 nm, FDHM = 1.1 ns,

FWHM = 27 nm, ω = 100 KHz) and the emission was observed in a 90° geometry relative to excitation at 495 nm with a photomultiplier tube (PMT). The electronic signal was processed digitally resulting in histograms representing the fluorescence decay curves of the samples. The response function of the LED was detected separately at 450 nm close to λ_{max}.

Analysis of TCSPC data. The duration of the excitation pulse of the LED (FDHM = 1.1 ns) cannot be neglected when determining fluorescence lifetimes in the ns time range via TCSPC. Therefore, the measured data correspond to the fluorescence decay function, *f(t)*, convoluted with the response function of the LED, *g(t)*.

$$f(t) = \int_{-\infty}^{t} g(t') f_{model}(t-t') dt' = \sum_{i=1}^{N} A_i \int_{-\infty}^{t} g(t') \exp\left(-k_i(t-t')\right) dt' \qquad (2)$$

Analysis of these data was performed using home-written software, which uses a sum of N exponential functions as model function $f_{model}(t)$. The square deviation between $f(t)$ and the data is minimized, taking the experimental instrument response for $g(t)$. The program makes efficient use of the fact that all data points are equidistant. The result of the fit are amplitudes A_i and rate constants k_i.

Ultra-High-Performance Liquid Chromatography (UPLC) analyses. For the separation of protein bound flavin molecules we adapted a previously described HPLC protocol (1) for Ultra-High-Perfomance Liquid Chromatography (UPLC). In brief, protein bound flavins were released by heat denaturation by incubation of the protein solution for 20 minutes at 99 °C. Precipitated protein was removed by centrifugation at 14.000 rpm for 20 minutes at room temperature. Subsequently the supernatant was applied to a Microcon YM3 centrifugal concentrator (13,000 rpm, 1 h, Millipore, Billerica, MA, molecular weight cutoff 3.000 Da) and the filtrate was analysed by UPLC. A UPLC system from Waters (Acquity UPLC H class; Waters, Milford, MA, USA) was used, employing a Titan C18 (5 cm x 2.1 mm, 1.9 µm particle size) column (Supelco, Bellefonte, PA, USA). The column was equipped with a pre-column of the same material, and 50 mM ammonium acetate, pH 6 (eluent A) and 70 % acetonitrile in A (eluent B) were used as solvents. The column temperature was set to 40 °C. Authentic FMN, flavin-adenine dinucleotide (FAD), riboflavin and lumichrome (all purchased from Sigma-Aldrich, St. Louis, MO) were used as reference compounds. Spectra of the eluted samples were recorded during the separation by a diode array detection system (PDA e λ detector, Waters, Milford, MA, USA). A solvent gradient (t = 0, 10:90 B:A, t = 0.5

- 2.39 min, 35:65 B:A, t = 2.39 – 2,42 min, 40:60 B:A, t = 2.42 – 3.07 min, 40:60 B:A, t= 3.07 - 3.1 min, 10:90 B:A, t = 3.1 - 5 min, 10:90 B:A) was applied using a constant flow of 0.605 ml/min.

Singular value decomposition (SVD) and deconvolution of time sequences of the steady-state UV/Vis spectra of iLOV-Q489D at pH 7.2. The data from bleaching and recovery experiments consist of a sequence of spectra measured at different times. These data were arranged into a matrix **D** of size N by M (with M ≤ N) and analyzed by singular value decomposition (SVD),

$$\mathbf{D} = \mathbf{U}\mathbf{W}\mathbf{V}^{\dagger} = \sum_{k=1}^{M} \mathbf{u}^{(k)} w_k \mathbf{v}^{(k)\dagger} \tag{2.1}$$

Here the orthogonal and normalized vectors **u** and **v** are the columns of the matrices **U** and **V** and form a basis in the space of spectra and concentration time profiles, respectively. The matrix elements w_k of the diagonal matrix **W** are positive and arranged in descending order. Subsequently, a sequence of approximations $A^{(K)}$ to the data matrix is formed

$$\mathbf{A}^{(K)} = \sum_{k=1}^{K} \mathbf{u}^{(k)} w_k \mathbf{v}^{(k)\dagger} \quad ; \quad K = 1, 2, 3... \tag{2.2}$$

and compared to the original data matrix

$$\sigma^{(K)} = \sqrt{\| \mathbf{D} - \mathbf{A}^{(K)} \| / NM} \tag{2.3}$$

A plot of $\sigma^{(K)}$ vs K indicates how many components K make a significant contribution to the data. In all our experiments we found K = 2, with the third components contributing less than 1 % of the first two.

The reduced data matrix $\mathbf{A}^{(2)}$ was then decomposed into the product of two spectra and two concentration time profiles,

$$\mathbf{A}^{(2)} = \mathbf{S}\mathbf{C}^{\dagger} = \mathbf{U}\mathbf{W}^{(2)}\mathbf{X}\mathbf{Y}\mathbf{V}^{\dagger} \tag{2.4}$$

Here, **S** is a matrix with two columns containing the species spectra, **C** is a matrix with two columns containing the concentration-time profiles of the two species, $\mathbf{W}^{(2)}$ contains only the first two diagonal elements of the matrix **W**, and **X** and **Y** are 2x2 matrices with **XY** = **1**. Since only one species (i.e. the neutral radical) absorbs at 615 nm, we take the corresponding row of $\mathbf{A}^{(2)}$ as the concentration profile of this species, i.e.

$$\mathbf{c}^{(2)} = \alpha \mathbf{A}^{(2)}(\lambda_{615}, t) \tag{2.5}$$

Were α is a scaling factor that determines the yield of the reaction at the final time. The concentration of the other species (i.e. the oxidized flavin) is defined by $c^{(1)} + c^{(2)} = 1$. The matrix **Y** is then calculated according to

$$\mathbf{C}^{\dagger} = \mathbf{Y}\mathbf{V}^{\dagger} \quad \rightarrow \quad \mathbf{Y} = \mathbf{C}^{\dagger}\mathbf{V} \tag{2.6}$$

Due to the orthogonality of the matrix **V**. Inversion of **Y** gives the matrix **X** and the species spectra according to

$$\mathbf{S} = \mathbf{U}\mathbf{W}^{(2)}\mathbf{X} \tag{2.7}$$

The only adjustable parameter in this procedure is the scaling factor α. If α is too small, the calculated spectrum of species 2 will have negative intensity in the region of strong absorption of species 1, if α is too large, the peaks of species 1 will also appear in the spectrum of species 2.

Molecular dynamics (MD) simulations. All MD simulations were performed as described previously for parental iLOV and iLOV-Q489K (2). Starting from the X-ray crystal structure of parental iLOV (PDB-code: 4EES, 1.8 Å (3)), a structural model of the iLOV-Q489D variant was constructed in YASARA Structure version 13.9.8 (4) using the YASARA-FoldX plugin (5) and employing the FoldX method (6). In order to explore alternative conformations of Q489D and the surrounding side chains below 6 Å distance from residue Q489, 10 independent runs including rotamer search employing a probability-based rotamer library were performed during the FoldX energy minimization. From the analysis of stabilization energy and interaction energies the most stable conformer was chosen as starting structures for MD simulations of iLOV-Q489D variant. The details of the FoldX prediction can be found elsewhere (2).

Molecular dynamics (MD) simulations were performed using the AMBER14 program.(7) The AMBER99SB force field (8, 9) parameters for the protein were augmented by the general AMBER force field (GAFF) (10) parameters for flavin mononucleotide (FMN) with the AMBER ff99 compatible RESP partial charges suggested in our previous work (2). The protonation states of titratable residues were assigned on the basis of pKa prediction carried out by using the PROPKA 3.1 program (11) and visual inspection. His471 and His495 were treated as HIE (NE2 protonated) and HIP (both ND1 and NE2 protonated), respectively. Side

chains of Asn and Gln residues were checked for possible flipping. Hydrogen atoms were added employing the tleap module of AmberTools14.(7) Crystal water molecules were kept. We performed the MD simulations with two possible different protonation states of the D489, either the protonated or deprotonated state. The phosphate group of flavin was deprotonated, carrying a charge of -2, which leads to a total charge of -4e/-5e for protonated or deprotonated D489 variant. The protein was solvated in an octahedral TIP3P (12) water box centered at the center of mass to ensure a water layer of 12 Å around the protein and neutralized by replacing solvent water molecules that were at least 5.5 Å away from any protein atoms by the sodium ions. The systems contained ~ 81,000 atoms in total, including ~26,500 TIP3P (12) water molecules.

In all MD simulations, long range electrostatic interactions were computed by using the Particle Mesh Ewald (PME) (13) method. To calculate the electrostatic interactions a cutoff of 10 Å with periodic boundary conditions was used. Initially, the solvent and the ions followed by the whole system were subjected to minimization using 10,000 steps of steepest descent followed by 3,000 steps of conjugate-gradient minimization. Following the energy minimization, the system was then slowly heated from 0 to 300 K for 50 ps. After heating, the systems were equilibrated for 1000 ps at 300 K. Finally, three independent MD simulations for the NPT ensemble at 300 K (for 50 ns) were carried out for iLOV-Q489D in each of the protonation states of D489 with a 2 fs integration step and a total length of 50 ns. No restraints were imposed on the coordinates in the simulations. VMD (14), Pymol (15), and AmberTools 14 (7) were used for molecular visualizations of the results and analysis of MD simulations. The detailed MD protocol was published elsewhere (2).

Geometric cutoff for hydrogen-bond distance and angle values of 3.2 Å and 150° were used, respectively. Cluster analysis of the structure of iLOV-Q489D along MD trajectories was performed by the GROMACS 4.5 (16) g_cluster tool and linkage method based on the side-chain conformation of D489 and the residues in FMN binding pocket with 1.35 Å RMSD as cutoff.

Supplementary Data

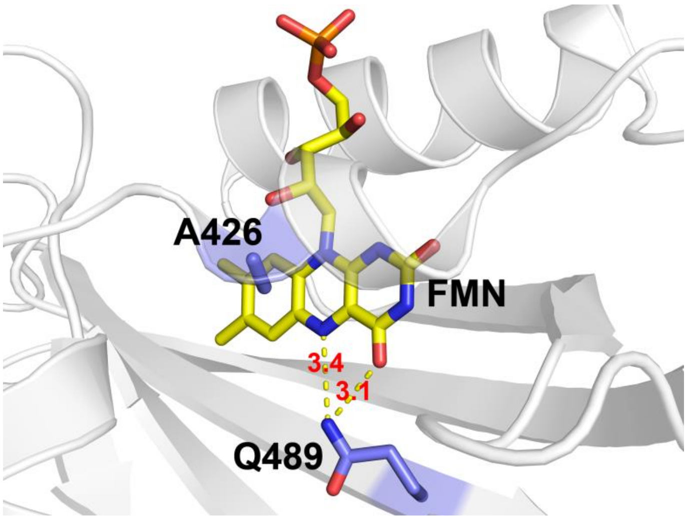

Figure S1: Close-up view of the FMN binding pocket of iLOV (PDB: 4EES) in cartoon representation. The FMN molecule, the Q489 side chain and A426 which in iLOV occupies the position of the photoactive cysteine are shown in stick representation. Nitrogen, oxygen and phosphorous atoms are shown in blue, red and orange, respectively. Carbon atoms are shown in blue (Q489, A426) or yellow (FMN). Dashed lines highlight relevant interatomic distances (labelled in red) between Q489-NE2 … FMN-O4 and FMN-N5, respectively.

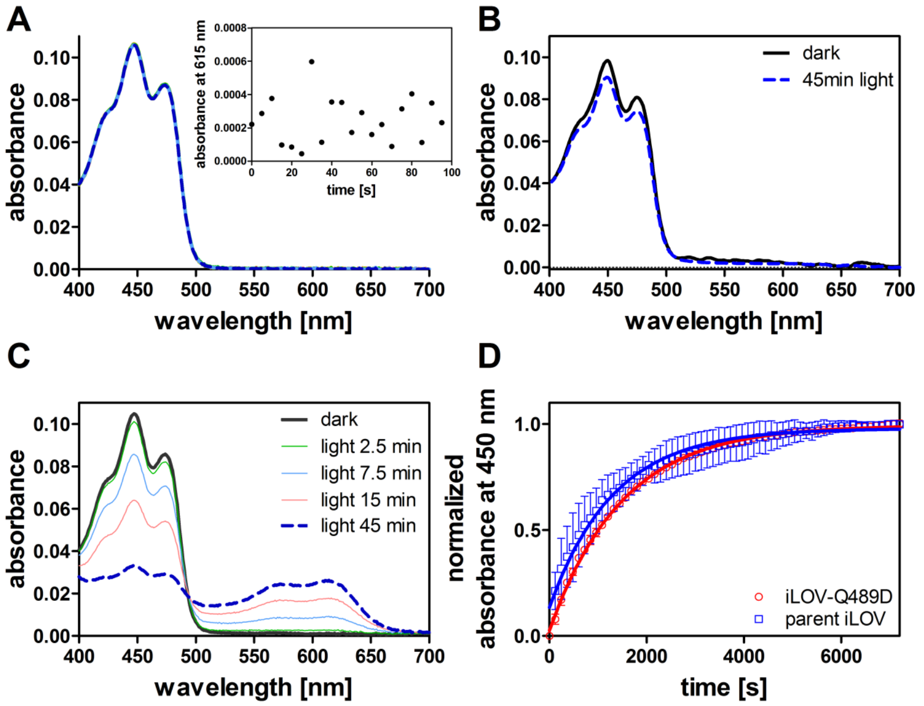

Figure S2: Photoreduction of parental iLOV in the presence (A) and absence of oxygen (B). Photoreduction in the presence of oxygen was carried out under identical conditions as described for iLOV-Q489D. To monitor photoreduction in the absence of oxygen, a protein solution was degassed by bubbling the solution for 15 minutes with Argon in a 1 cm quartz cuvette closed with a rubber septum. The corresponding degassed solution was illuminated for up to 45 minutes without any detectable formation of $FMNH^{\bullet}$. (C) Photoreduction of parental iLOV in a degassed solution containing the sacrificial electron donor EDTA (C). Photoreduction of parental iLOV is only possible in the absence of oxygen and the addition of excess amounts of the sacrificial electron donor EDTA. To enforce photoreduction of parental iLOV, the protein sample (6.5 µM) was mixed with approx. 150-fold excess EDTA (1 mM) and the solution was degassed by bubbling Argon for 15 minutes through the solution in a quartz cuvette closed with a rubber septum. The resulting solution was illuminated for up to 45 minutes. All measurements were carried out in in 200 mM sodium phosphate buffer pH 7.2 supplemented with 10 mM NaCl. (D) Reoxidation kinetics of iLOV-Q489D (red circles) and parental iLOV (blue rectangles) after photoreduction. iLOV-Q489D was photoreduced in the presence of oxygen as shown in Figure 2, A of the main manuscript. Subsequently, reoxidation of the flavin chromophore was monitored for 2 hours in the dark and recording sequential spectra. Parental iLOV was photoreduced as described in panel C and subsequently flavin reoxidation was monitored for 2 hours in the dark. Reoxidation efficacy was quantified by plotting the normalized rise in absorbance at 450 nm against the incubation time in the dark. Experimental data was fit using a single exponential decay function (blue and red solid line) to obtain the reoxidation lifetime τ_{FMNox} = 1502 ± 27 s (iLOV-Q489D) and τ_{FMNox} = 1376 ± 520 s (parental iLOV). Please note that the error associated with the reoxidation measurement of parental iLOV is quite large probably due to the overlay of oxygen diffusion and reoxidation. Additionally, the degassing of the protein solution within the cuvette results in variable volume loss further complicating the measurement. Error bars correspond to the standard deviation of the mean derived from three independent measurements.

pH-dependence of iLOV-Q489D photoreduction

One possible explanation for low photoreduction yields at low acidic and high basic pH values could be a pH-dependent unfolding of iLOV-Q489D during the photoreduction measurement, which would result in the release of the flavin chromophore from the protein, hence abolishing the possibility of photoreduction by the protein. At a pH < 4, the corresponding UV/Vis spectra (Figure S3, A, B and Figure S4, A, B) show evidence for unfolding of iLOV-Q489D during the photoreduction measurement, i.e. the vibronic fine structure with a maximum at around 470 nm is lost during the photoreduction measurement and the whole spectra are broadened which results in a specific rise in absorbance at 495 nm (see Figure S4, A and B). At all other pH values no evidence for unfolding of iLOV-Q489D during the measurement is observed (see Figure S3, C-J). To quantify the pH-dependent unfolding of iLOV-Q489D at the two lowest pH values (pH 2.6 and pH 3.0), we measured sequential UV/Vis spectra under identical conditions as used in the photoreduction experiment but did not illuminate the sample (Figure S4, A and B). As control, the pH stability of iLOV-Q489D at the two highest pH values (pH 9.0 and 10.6) was measured (see Figure S4, C and D). At pH 2.6 (Figure S4, A) and pH 3.0 (Figure S4, B) the UV/Vis spectra are indicative of unfolding and release of the flavin chromophore of iLOV-Q489D. The unfolding and flavin release proceeds with a lifetime of τ_{pH}= 148 ± 9 seconds at pH 2.6 and τ_{pH} = 191 ± 11 seconds at pH 3.0. This corresponds to an illumination time of about 25 and 32 seconds at pH 2.6 and pH 3.0, respectively. Thus the very low photoreduction yields observed below pH 4.0 can readily be explained by pH-dependent unfolding of iLOV-Q489D which results in the release of the flavin chromophore from the protein thereby abolishing photoreduction. In contrast, at basic pH values, iLOV-Q489D appears stable (Figure S4, C and D). Hence the low photoreduction yields at basic pH values cannot be attributed to unfolding of the protein.

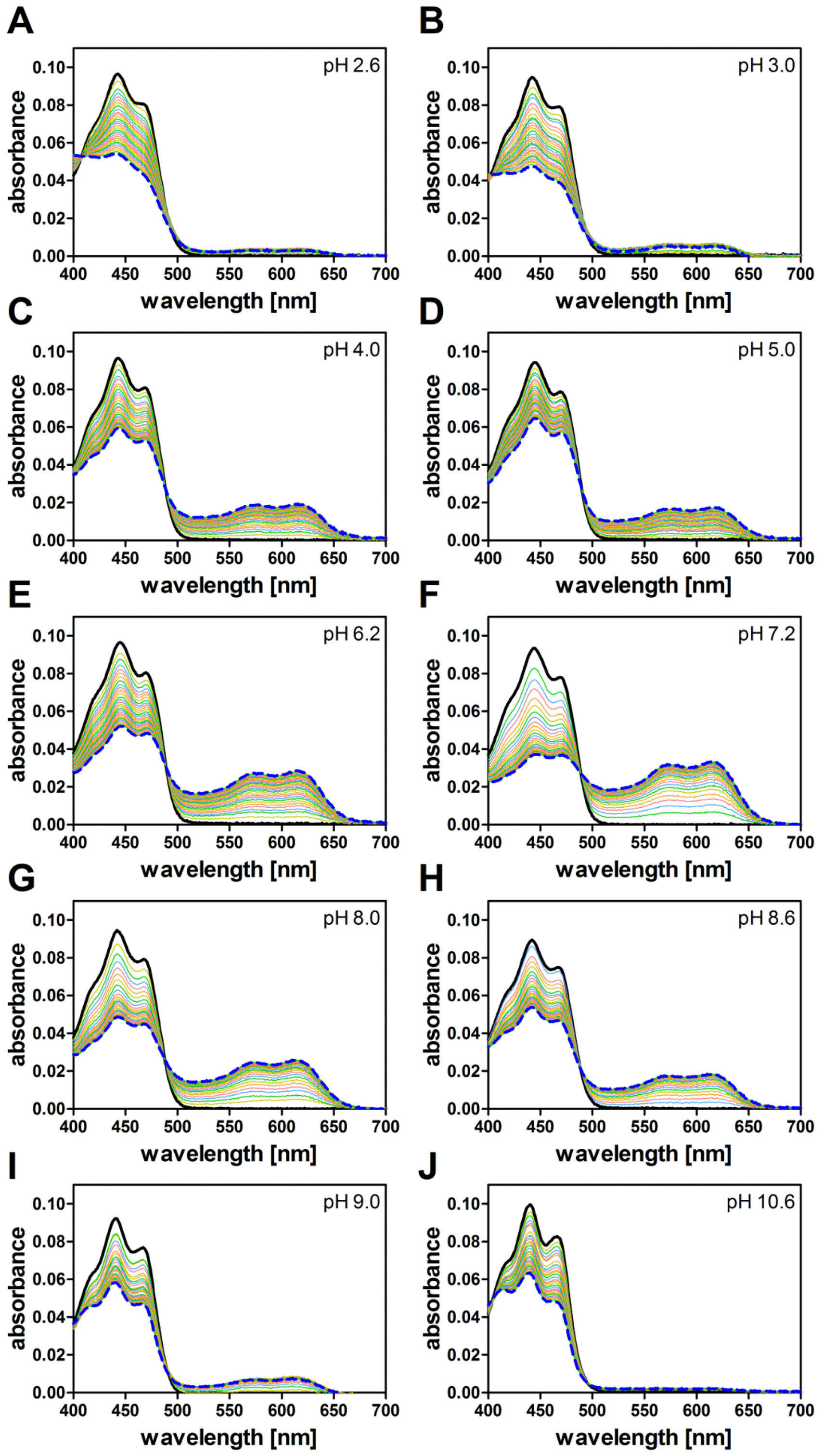

Figure S3: Spectral changes associated with illumination of iLOV-Q489D at pH 2.6 to pH 10.6. For clarity only one exemplary measurement out of three independent experiments per pH value is shown.

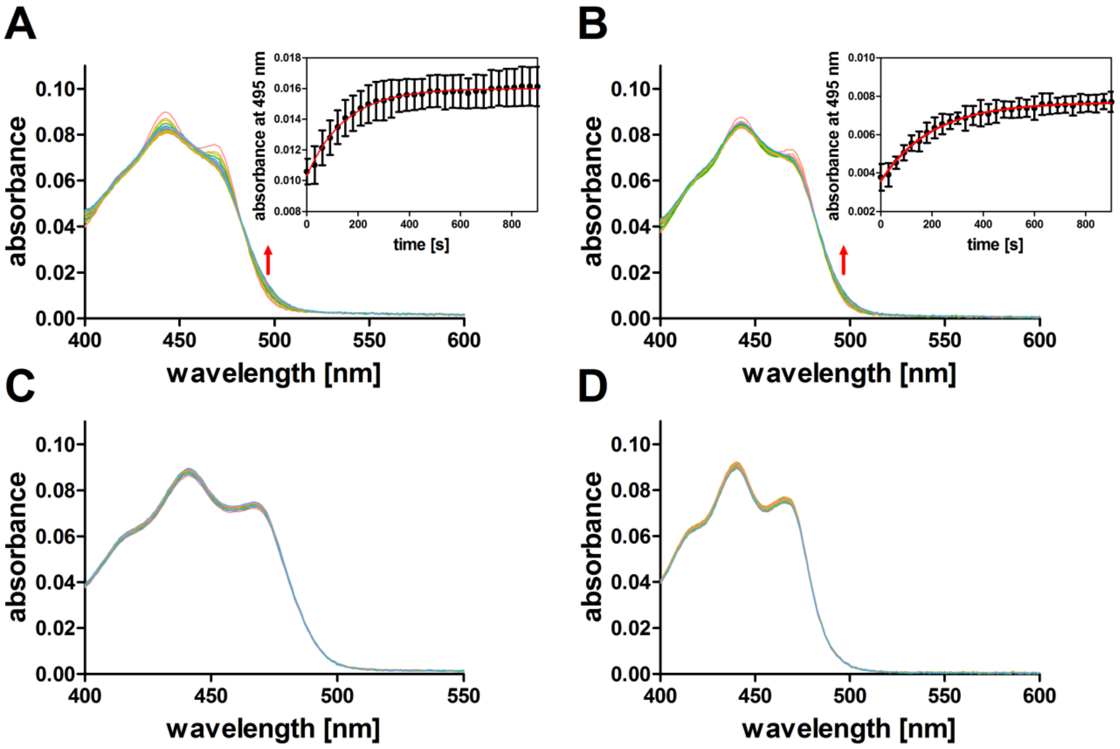

Figure S4: pH stability of iLOV-Q489D at pH 2.6 (A), pH 3.0 (B), pH 9.0 (C) and pH 10.6 (D). iLOV-Q489D was diluted to an OD_{450nm} of approx. 0.1 in either 200 mM phosphate/citrate buffer (pH 2.6 and pH 3.0) supplemented with 10 mM NaCl or in 200 mM Glycine/NaOH buffer (pH 9.0 and pH 10.6) supplemented with 10 mM NaCl. The samples were placed in a 1 cm quartz cuvette and incubated at 25 °C in the spectrophotometer for up to 15 minutes. Sequential UV/Vis spectra were recorded at a time interval of 30 sec (shown in rainbow coloring). At pH 2.6 (A) and pH 3.0 (B) the UV/Vis spectra are indicative of unfolding and the release of the flavin chromophore of iLOV-Q489D, which was followed over time by plotting the rise in absorbance at 495 nm (red arrow) against the incubation time (inset). The resulting data could be fitted using a single-exponential decay function. The unfolding proceeds with a lifetime of τ_{pH}= 148 ± 9 seconds at pH 2.6 and τ_{pH}= 191 ± 11 seconds at pH 3.0. This corresponds to an illumination time of about 25 and 32 seconds at pH 2.6 and pH 3.0, respectively. At pH 9.0 (C) and pH 10.6 (D) no spectral changes are observed, which suggests that at those pH values iLOV-Q489D is stable during the incubation time. Error bars correspond to the standard deviation of the mean derived from three independent measurements.

Spectral changes associated with photoreduction of iLOV-Q489D at pH 7.2, pH 2.8 and pH 9.8

At pH 7.2 the respective spectra (Figure S3, F and Figure S5, A) provide clear evidence for the formation of $FMNH^{\bullet}$, with the process being fully reversible in the dark (Figure S5, B). We performed singular value decomposition (SVD) of this data set (Figure S6). The plot of $\sigma^{(K)}$ vs K (Figure S6, A, inset) indicates, that only two components contribute significantly to the spectra at pH 7.2. We considered the absorption in the range 250 – 700 nm, and up to 290 s. Applying a two component model to the data yields the reconstructed spectra in the range $0.8 < \alpha < 0.9$ and the concentration time profiles for $\alpha = 0.85$ of FMN_{ox} and the product $FMNH^{\bullet}$ (Figure S6, A; B). Note that this reconstruction conserves the isosbestic points in the two spectra.

At pH 2.8 and pH 9.8 we observe an initial rapid formation of minor amounts of $FMNH^{\bullet}$, as evidenced by an initial increase of the $FMNH^{\bullet}$ absorbance band at 615 nm, which does not increase above a certain value. Interestingly, the band corresponding to FMN_{ox} (at 450 nm) does decrease further after prolonged illumination (Figure S5, C and E). This is accompanied with the formation of a new maximum at around 390 nm (Figure S5, C and E). Hereby the initially formed $FMNH^{\bullet}$ is slowly further interconverted, as evidenced by the loss of absorbance at 615 nm during prolonged illumination (Figure S5, C and E, inset). In both cases the process appears irreversible in the dark (Figure S5, D and F). To address this issue we repeated the experiment and subsequently extracted the protein-bound flavins from the protein solution and analyzed the flavin composition by Ultra-High-Performance-Liquid-Chromatography (UPLC) (Figure S7). Native (pH 7.2) iLOV-Q489D binds predominately FMN with minor amounts of lumichrome present in the sample (Figure S7, C, D). Since lumichrome is a photodegradation product of flavins (17) it seems likely, that during sample preparation, which was carried out under daylight conditions, a certain amount of lumichrome accumulated. In contrast, samples illuminated at pH 2.6 and 9.8 exclusively contain luminchrome after prolonged illumination (Figure S7, E-H). Thus the observed decrease of the FMN_{ox} absorbance band during prolonged illumination appears to be related to photodamage of the flavin chromophore.

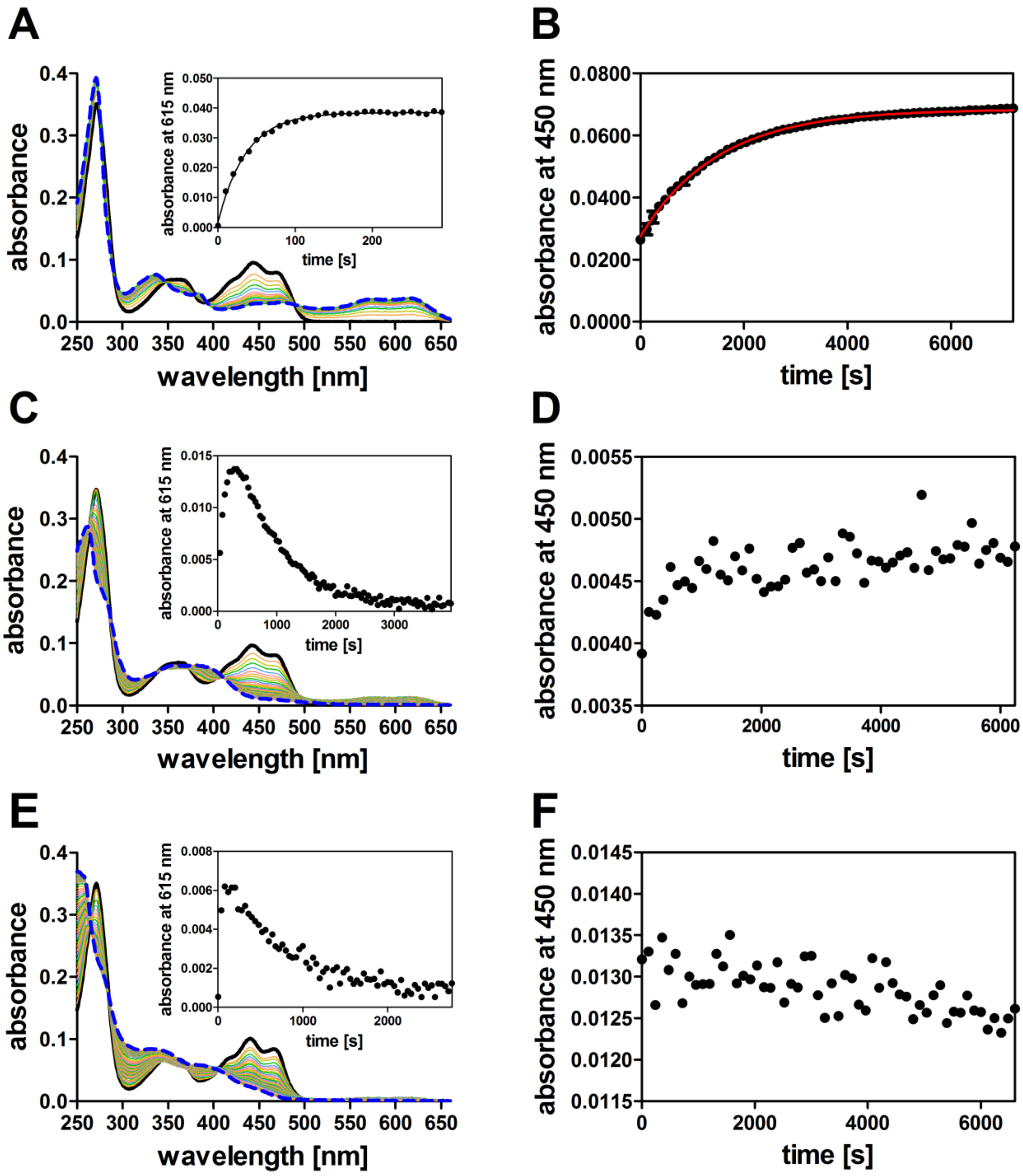

Figure S5: Photoreduction of iLOV-Q489D and FMN reoxidation at pH 7.2 (A, B), pH 2.8 (C, D) and pH 9.8 (E, F). The dark-adapted sample (solid black line) was illuminated and sequential spectra were recorded (rainbow coloring, illumination time increment: 10 s) until no further spectral changes did occur (dashed blue line). The inset shows the absorbance trace at 615 nm indicative of $FMNH^{\bullet}$ formation and conversion. After complete photoreduction, the samples were incubated in the dark and sequential spectra were recorded (data not shown). As marker for FMN reoxidation the absorbance at 450 nm was plotted against incubation time (B, D, F). Only at pH 7.2 complete FMN reoxidation is observed which proceeds with a lifetime of τ_{FMNox} = 1502 ± 27 s. The respective data was fit to a single exponential decay function (B; red line). Error bars correspond to the standard deviation of the mean derived from three independent measurements.

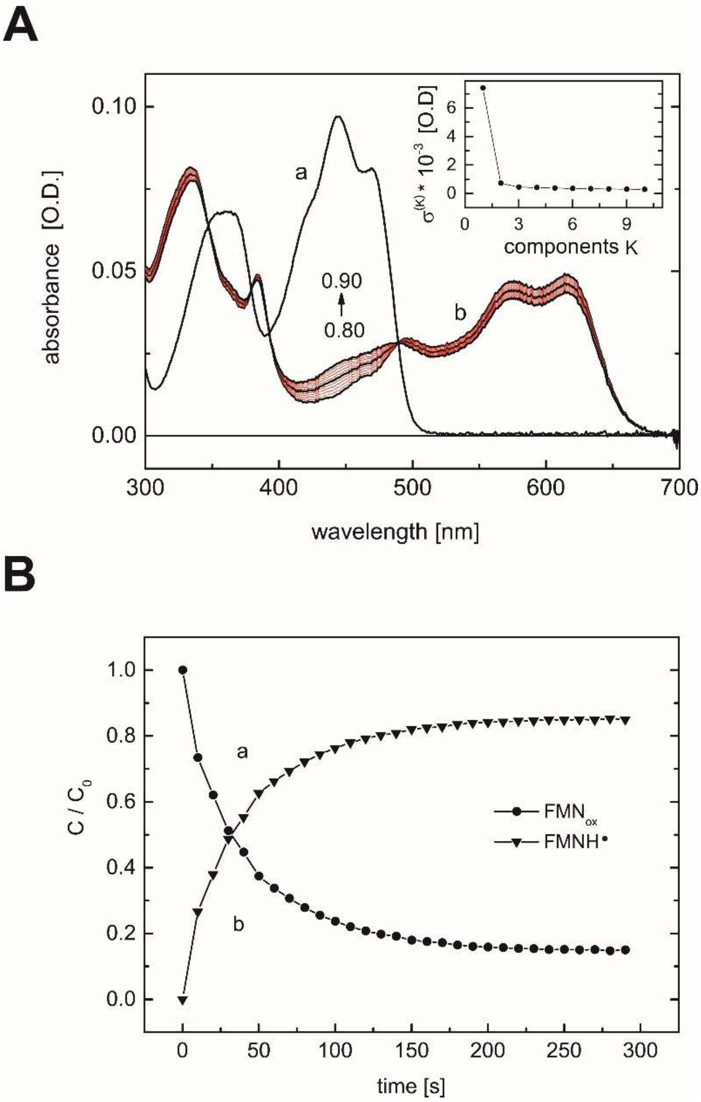

Figure S6: SVD analysis of the photoreduction data of iLOV-Q489D at pH 7.2 (data from Figure S5, A). (A): The reconstructed spectra of FMN_{ox} (a) and $FMNH^{\bullet}$ (b) in the range 0.8 < α < 0.9. The inset shows $\sigma^{(K)}$ as a function of the components, K, indicating that two significant components are present. (B): Concentration time profiles for α = 0.85 of FMN_{ox} (a) and $FMNH^{\bullet}$ (b).

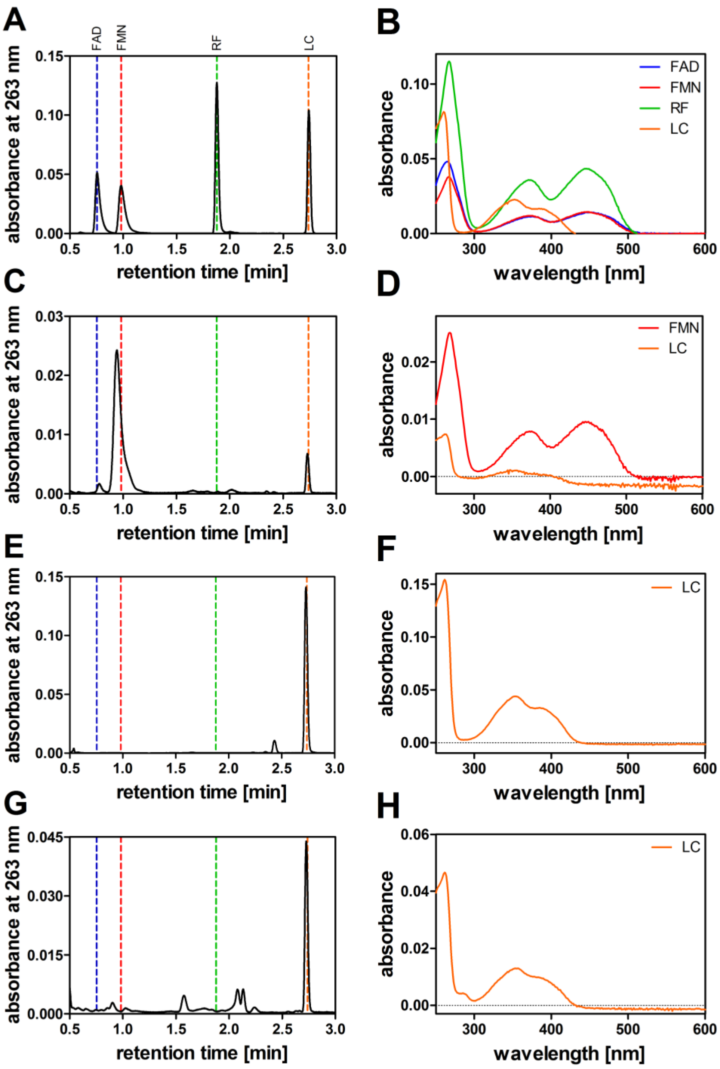

Figure S7: UPLC analysis of iLOV-Q489D at pH 7.2 and after photoreduction at pH 2.6 and 9.8. Authenic flavin adenine dinucleotide (FAD), flavin mononucleotide (FMN), riboflavin (RF) and lumichrome (LC) were used as standards. The corresponding UPLC chromatogram (A) is shown along with the UV/Vis spectrum of the respective elution peak (B). FAD, FMN, RF and LC elute at 0.75 min, 0.97 min, 1.87 min and 2.73 min, respectively (marked by dashed lines). The pH of the protein samples was adjusted by dilution (1:10) with 200 mM sodium phosphate buffer pH 7.2, 200 mM sodium phosphate /citrate buffer pH 2.6 or 200 mM glycine/NaOH buffer pH 9.8, respectively. All buffers were supplemented with 10 mM NaCl. The native chromophore content of iLOV-Q489D was determined by extracting the flavins from a dark adapted sample adjusted to pH 7.2 (chromatogram: C, elution peak spectra: D). At pH 2.6 and pH 9.8 the samples were illuminated until no further spectral changes occurred (60 min) and protein bound flavins were extracted as described in the Supporting Methods section. Subsequently, the extracted flavins were analysed by UPLC. The corresponding chromatograms and elution peak spectra are shown in panel E, F (pH 9.8) and G, H (pH 2.6), respectively. Please note, that at pH 2.6 the overall signal intensity was 3-fold lower than for the pH 9.8 sample, although similar amounts of protein were denatured to release the protein bound flavins.

UV/Vis spectra of iLOV-Q489D along with computational predictions suggest that the newly introduced Asp is protonated at neutral pH values. Alteration of the H-bonding network around the FMN-N5 atom in iLOV, e.g. by substitution of Q489 for Asp or by the presence of either a protonated or unprotonated (and hence charged) Asp side chain in iLOV-Q489D at different pH values, is expected to influence the flavin absorbance band in the violet/blue region of the spectrum (2). To address this issue we compared UV/Vis spectra of parental iLOV and iLOV-Q489D at pH 7.2, pH 2.8 and pH 9.8 (Figure S8).

Parental iLOV (Figure S8, A, black dashed line) shows an absorbance maximum at around 447 nm (corresponding to the S0-S1 transition). At pH 7.2 (red solid line) and pH 2.8 (green solid line) the absorbance maximum of iLOV-Q489D is slightly blue shifted by 4 or 5 nm, respectively. In contrast, at pH 9.8 the absorbance maximum of iLOV-Q489D is clearly blue shifted by 7 nm to about 440 nm. To study how the Q489D substitution influences the structure of iLOV, the iLOV-Q489D variant was constructed *in silico*, starting from the parental iLOV crystal structure, and a rotamer search was conducted with FoldX exploring alternative conformations of the surrounding side chains. To investigate possible conformational changes associated to the introduced Q489D mutation, we performed three independent 50 ns MD simulations of the iLOV-Q489D variant starting from the most stable FoldX predicted structure in both protonated and deprotonated states of D489. Analysis of the D489 side chain highlights the structural changes surrounding residues D489, near to FMN chromophore during MD simulations (Figure S8, B and C). Figure S8, C shows the analysis of the interatomic distances for the D489-FMN interaction along MD trajectories, indicating that protonated D489 preserves an H-bond with the FMN-O4 atom. Independent H-bond analysis of the trajectories shows that in ~70% of the trajectories protonated D489 forms an H-bond with FMN-O4. In contrast, in the simulation containing deprotonated D489 the D489 side chain samples multiple conformations along the trajectories but does at no time form an H-bond to the FMN chromophore. Inspection of the simulation reveals that D489 flips away from the FMN chromophore followed by the entrance of some water molecules to the FMN binding pocket (Figure S8, B and C). Detailed information about the tree independent MD simulations can be found in Figure S16-S21. The 7 nm blue-shift observed for iLOV-Q489D at pH 9.8 can readily be explained based on our MD simulation of iLOV-Q489D with deprotonated D489, where the side chain of D489 flips away from the chromophore (Figure S8, B and C), with the loss of H-bonding between D489 and FMN resulting in a blue-shifted absorption maximum similar to the recently characterized iLOV-Q489K variant (2). Likewise the small 4-5 nm blue-shift observed at pH 7.2 and pH 2.8, respectively, can be explained by

altered protein-FMN interactions compared to parental iLOV. In parental iLOV the NE2 atom of Q489 is in close proximity to the FMN-N5 atom (<3.5 Å) (2) and forms an H-bond with the FMN-O4 atom. While the H-bond to FMN-O4 is retained in our MD simulations of iLOV-Q489D with protonated D489, the average distance between the OD2 atom of D489 and FMN-N5 is increased (>4.5 Å) (Figure S8, C), which may result in a blue-shifted absorbance maximum.

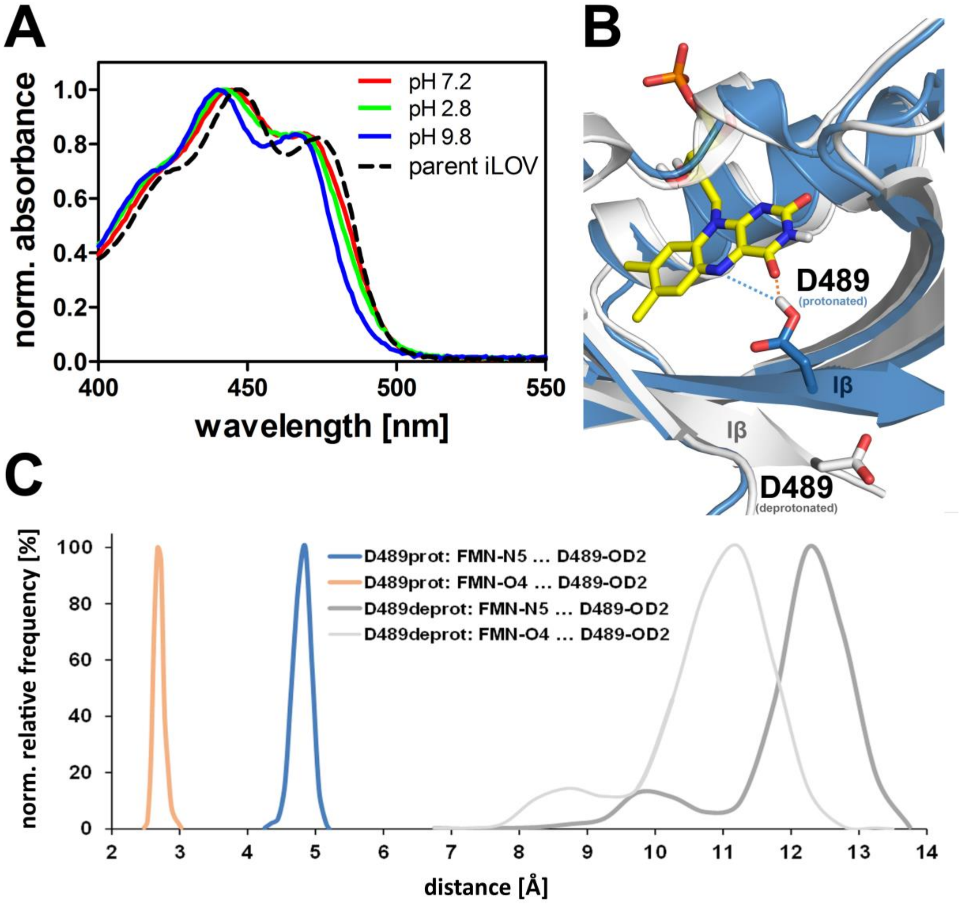

Figure S8: UV/Vis spectra of parental iLOV (black dashed line), iLOV-Q489D at pH 7.2 (red solid line), pH 2.8 (green solid line) and pH 9.8 (blue solid line) (A). Close-up view of the FMN binding pocket of iLOV-Q489D in cartoon representation (B). Representative structure of the FMN chromophore binding pocket of iLOV in protonated D489 and deprotonated D489 taken from the MD trajectories. The snapshot was selected based on cluster analysis of the MD trajectories. Average distance distribution curve of the interatomic distances for the indicated residues (in Å) calculated over three independent MD trajectories of iLOV-Q489D with protonated and deprotonated D489 (C). H-bond analysis (with geometric cutoff for hydrogen-bond distance and angle values of 3.2 Å and 150°, respectively) shows that D489-OD2 preserves a hydrogen bond with FMN-O4 ~70% of the time in the simulations with protonated D489. The depicted distance distribution curves were derived from the data shown in Figure S16, S18 (protonated D489) and Figure S17, S19 (deprotonated D489).

Generation of Trp• reference spectrum in solution. We excited FMN at 447 nm in the presence of Trp and measured TA on a 20 µs time window. The resulting difference spectrum is shown in Figure S9. The spectrum remained unchanged from 9 - 20µs and was averaged. The spectral signature exhibits similarity to the DADS2 of iLOV-Q489D (Figure 3, C) and the contributing species can be assigned to FMNH• and Trp• [43]. An identical measurement was performed with FMN and Cys, also resulting in the formation of FMNH• and the concomitant CysS•. Since the cysteinyl radical does not absorb in the visible range [44], the difference between FMN+Trp and FMN+Cys yields the spectrum of Trp• (Figure S9, inset).

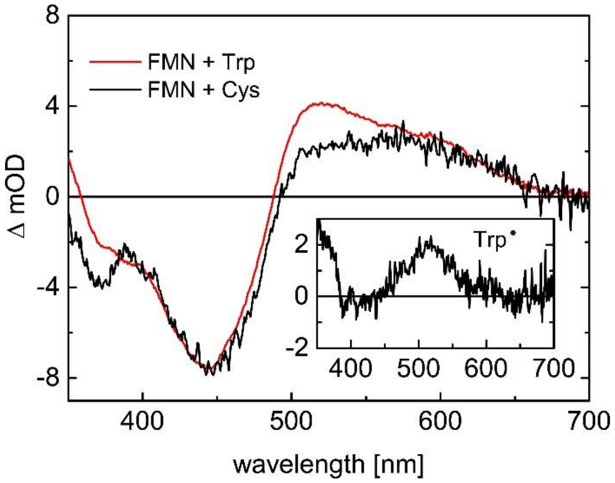

Figure S9: Comparison of the difference absorption spectra of FMN + Trp (red line) and FMN + Cys (black line) in the absence of protein. Blue-light excitation of FMN in the presence of Trp leads to the formation of FMNH• and Trp•. When exciting FMN in the presence of Cys, only the neutral flavin semiquinone radical FMNH• absorbs in the visible range of the spectrum. The spectrum of Trp•-TrpH (inset) was obtained as the difference of the two transient spectra scaled to the same minimum at 450 nm.

Electron paramagnetic resonance (EPR) spectroscopy reveals the presence of a stable radical pair in iLOV-Q489D

EPR spectra of the photoexcited FMN triplet state.

Figure S10 shows echo-detected EPR spectra recorded 500 ns after laser excitation of parental iLOV and iLOV-Q489D at 30 K. Besides the narrow central signal due to the photoaccumulated radical and radical pair products, the typical spectrum of the flavin excited triplet state is detected (18). The small changes of the fine structure parameters indicate the sensitivity of triplet electronic structure to the Q489D mutation in the vicinity of the flavin.

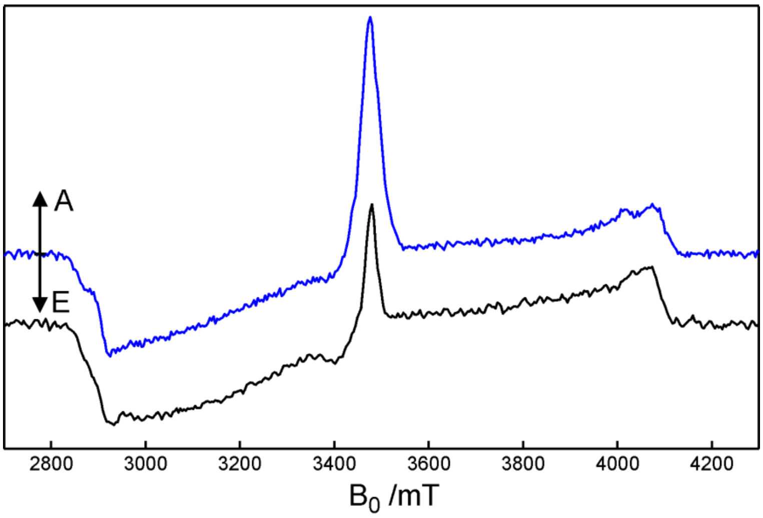

Figure S10: X-band echo detected EPR spectra of parental iLOV (black trace) and iLOV-Q489D (blue trace) detected 500 ns after 450 nm laser flash at 30 K using t_p-τ-2t_p Hahn echo sequence with t_p = 20ns and τ = 200ns.

Simulation of radical pair EPR spectrum.

The radical pair spectrum was analysed using the EasySpin toolbox for the Matlab program package (19, 20). Because the counter radical in the pair is unknown, the spectrum was calculated using inhomogeneous Gaussian linewidth of 3.0 mT. The best agreement between experimental and calculated spectra was found for |D| = 4.0 ± 1.0 mT and E=0 (see Figure S11).

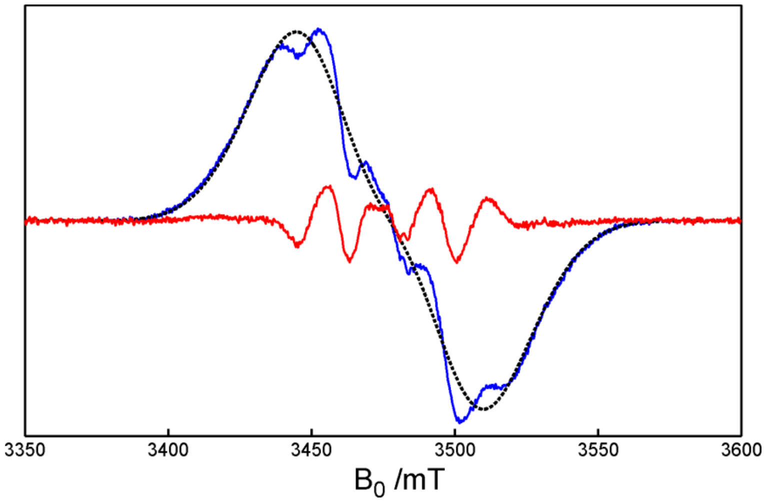

Figure S11: X-band continuous wave (cw) EPR spectra of iLOV-Q489D (blue trace) recorded after 20 min illumination of the dark adapted sample. The spectrum was corrected for $FMNH^{\bullet}$ contribution using the EPR spectrum recorded after sample annealing (see Figure 5 of the main manuscript). The dashed line shows the best fit using a numerical solution of the spin Hamiltonian accounting for fine (zero field) structure with |D| = 4.0 mT, E=0. The residual is shown by red line.

Experimental and computational analysis of the eT pathway in iLOV-Q489D

As potential electron donating redox active amino acids iLOV contains three tyrosines (Y416, Y459 and Y484) as well as one tryptophan (W467) (Figure S12).

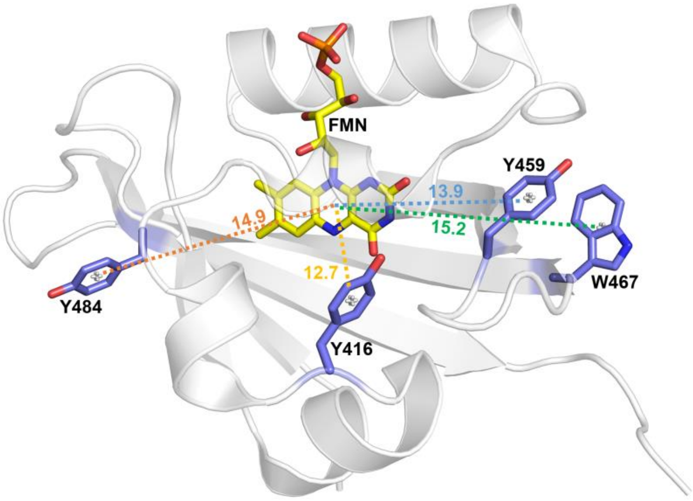

Figure S12: Potential electron donating Tyr and Trp residues in parental iLOV (A). Parental iLOV (PDB: 4EES) is shown in transparent cartoon representation with the FMN chromophore depicted as sticks with yellow carbon atoms. All tyrosine and tryptophan residues of parental iLOV are shown as sticks with carbon atoms in light blue. Nitrogen, oxygen and phosphorous atoms are shown in dark blue, red and orange, respectively. Dashed lines highlight the distance between the center of mass of the respective aromatic ring system and the center of mass of the FMN isoalloxazine ring. For clarity only center of mass distances between the amino acids and FMN are shown. All other measured distances can be found in Table S1 below.

Table S1: Center of mass (black) and edge-to-edge (red) distances between the redox active amino acids Y416, Y459, Y484, W467 and the FMN chromophore in iLOV. All values were derived from the iLOV X-ray structure (PDB-ID: 4EES)

	center of mass distance (Å)[§] / edge-to edge distance (Å)[$]				
	**FMN**	**Y416**	**Y459**	**Y484**	**W467**
**FMN**	-	12.7	13.9	14.9	15.2
**Y416**	9.9	-	11.2	28.1	10.5
**Y459**	9.1	8.8	-	21.8	18.8
**Y484**	10.2	20.3	26.1	-	29.3
**W467**	10.8	16.6	9.8	26.5	-

[§]: The center of mass of each group (the tricyclic isoalloxazine of the FMN, the indole part of Trp or the benzene ring of Tyr) was determined as the mean of the crystallographic coordinates of all the ring carbon and nitrogen atoms. The center of mass distance was then determined between the corresponding center coordinates. [$]: The edge-to-edge distance was determined as the smallest separation between C or N ring atoms of the respective groups.

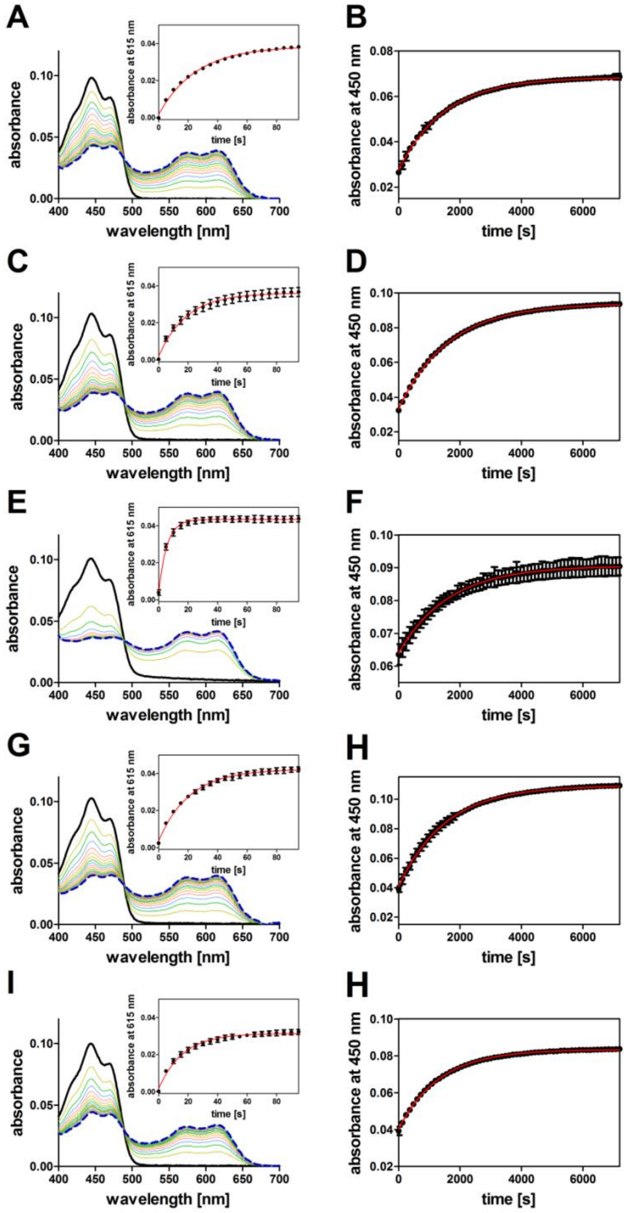

Figure S13: Photoreduction and reoxidation of iLOV-Q489D (A,B), iLOV-Q489D/Y416F (C,D), iLOV-Q489D/Y459F (E,F), iLOV-Q489D/Y484F (G, H) and iLOV-Q489D/W467F (I,J). The left five panels show the respective photoreduction measurements, with the inset depicting the time trace for FMNH$^{\bullet}$ formation extracted from the corresponding sequential UV/Vis spectra by plotting the absorbance at 615 nm against the illumination time. The right side of the figure shows the reoxidation time trace obtained from sequential UV/Vis spectra (not shown) by plotting the absorbance at 450 nm against the incubation time. A single exponential fit of the experimental data (red line) yields reoxidation lifetimes (τ_{FMNox}) of 1502 ± 27 s (iLOV-Q489D), 1621 ± 25 s (iLOV-Q489D/Y416F), 1622 ± 66 s (iLOV-Q489D/Y459F), 1525 ± 41 s (iLOV-Q489D/Y484F) and 1329 ± 29 (iLOV-Q489D/W467F), respectively. Error bars correspond to the standard deviation of the mean derived from three independent measurements. Photoreduction and reoxidation measurements were carried out as described in the Materials and Methods section at 25 °C in 200 mM sodium phosphate buffer pH 7.2 supplemented with 10 mM NaCl.

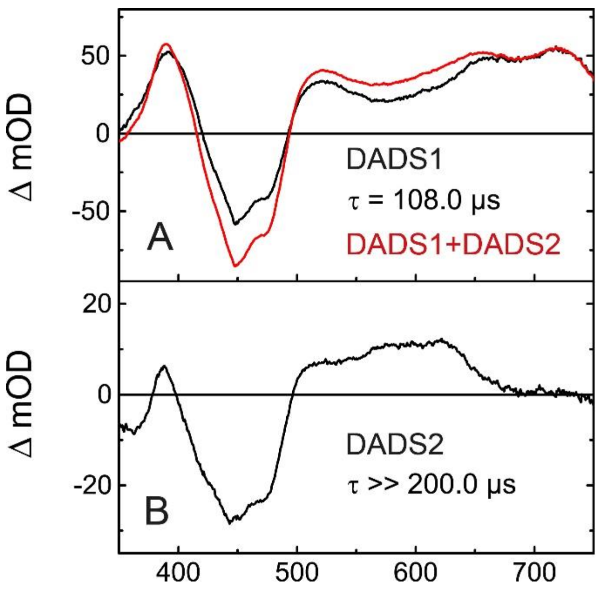

Figure S14: DADS of iLOV-Q489D/W467F obtained via global analysis of a 200 µs TA data set. (A) DADS1 (black line) associated with the excited triplet state of FMN decays with τ = 108.0 µs. The sum of both DADS (red line) representing the pure triplet spectrum of FMN incorporated in iLOV-Q489D/W467F is displayed for comparison. (B) DADS2 is non-decaying on the measured streak window of 200 µs and shows typical contributions of $FMNH^{\bullet}$

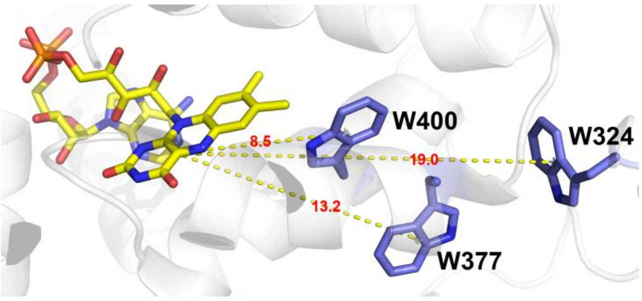

Figure S15: Close-up view of the crystal structure of *Arabidopsis thaliana* Cry1 (PDB: 1U3D). ArCry1 is shown in transparent cartoon representation with the FAD chromophore depicted as sticks with yellow carbon atoms. The tryptophan triad (W400, W377, W324) is shown as sticks with carbon atoms in light blue. Nitrogen, oxygen and phosphorous atoms are shown in dark blue, red and orange, respectively. Dashed yellow lines highlight the distance between the center of mass of the respective aromatic ring system and the FAD C4a atom. For clarity only center of mass distances between the amino acids and FMN are shown. The corresponding distances (in Å) are given in red.

Table S2: Center of mass (black) and edge-to-edge (red) distances between the components of the Trp triad in *Arabidopsis thaliana* Cry1. All values were derived from the AtCry X-ray structure (PDB-ID: 1U3D)

	**center of mass distance (Å) / edge-to-edge distance (Å)[§]**			
	**FAD**	**W400**	**W377**	**W324**
**FAD**	-	8.5	13.2	19.0
**W400**	4.5	-	7.3	11.0
**W377**	9.7	4.8	-	8.1
**W324**	14.7	8.7	5.1	-

[§]: distances according to Hore *et al.* (2009) (21)

Molecular dynamic (MD) simulations of iLOV-Q489D

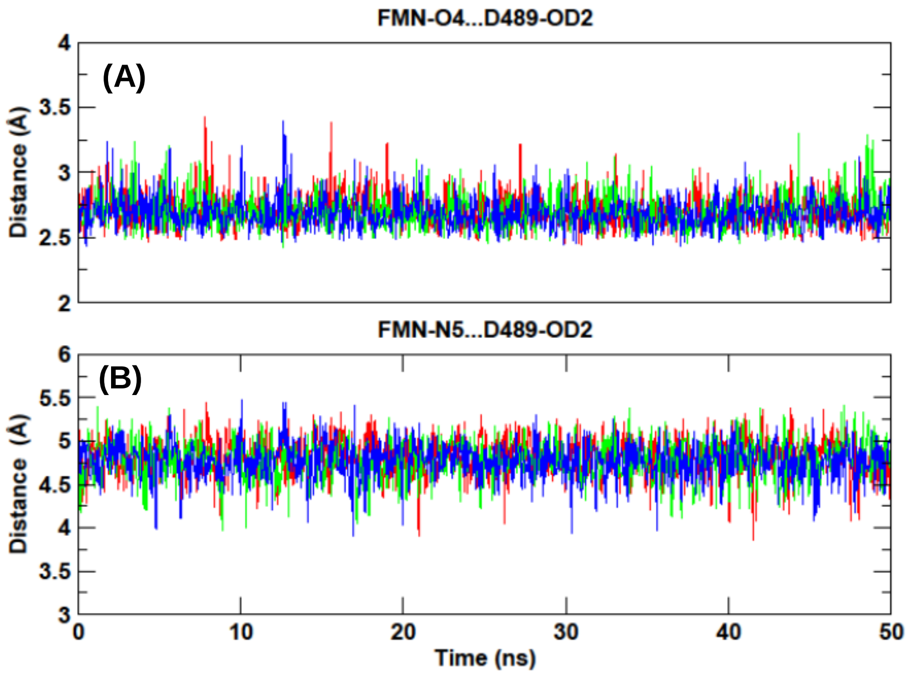

Figure S16: Interatomic distances for the indicated residues (atoms) pairs (in Å) calculated over the MD trajectories of iLOV-Q489D variant with protonated D489; (A) FMN-O4 and D489-OD2 distance, (B) FMN-N5 and D489-OD2 distance for three independent 50 ns MD runs (run_1, red; run_2, green; run_3, blue). H-bond analysis (with geometric cutoff for hydrogen-bond distance and angle values of 3.2 Å and 150°, respectively.) shows that D489-OD2 preserves a hydrogen bond with FMN-O4 in ~70% of the simulations time.

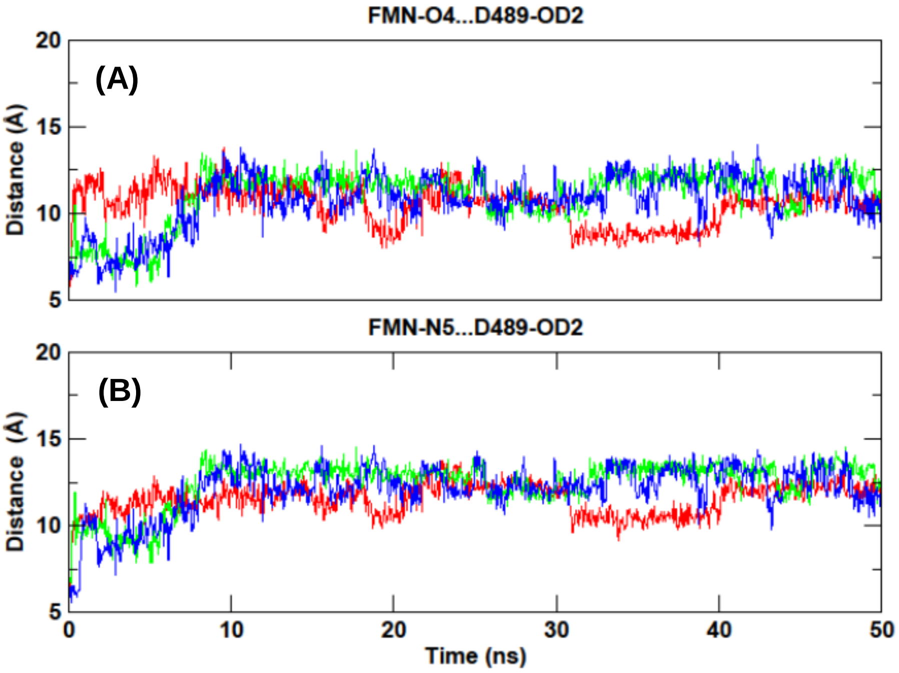

Figure S17: Interatomic distance for the indicated residue(atom) pairs (in Å) calculated over the MD trajectories of iLOV-Q489D variant with deprotonated D489; (A) FMN-O4 … D489-OD2 distance, (B) FMN-N5 … D489-OD2 distance for three independent 50 ns MD runs (run_1, red; run_2, green; run_3, blue).

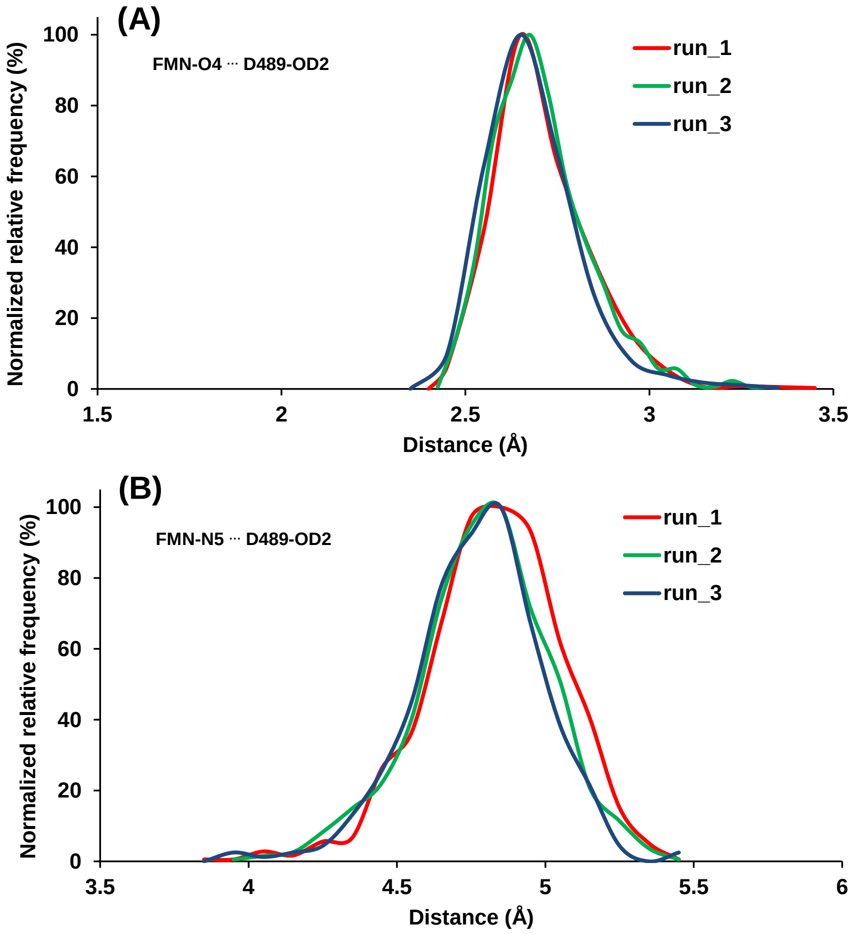

Figure S18: Distance distribution curve of the interatomic distances for the indicated residue (atom) pairs (in Å) calculated over the MD trajectories of iLOV-Q489D variant with protonated D489; (A) FMN-O4 … D489-OD2 distance distribution, (B) FMN-N5 … D489-OD2 distance distribution for three independent 50 ns MD runs (run_1, red; run_2, green; run_3, blue). The data was used to generate the distance distribution curves shown in Figure S8, C (orange and blue lines).

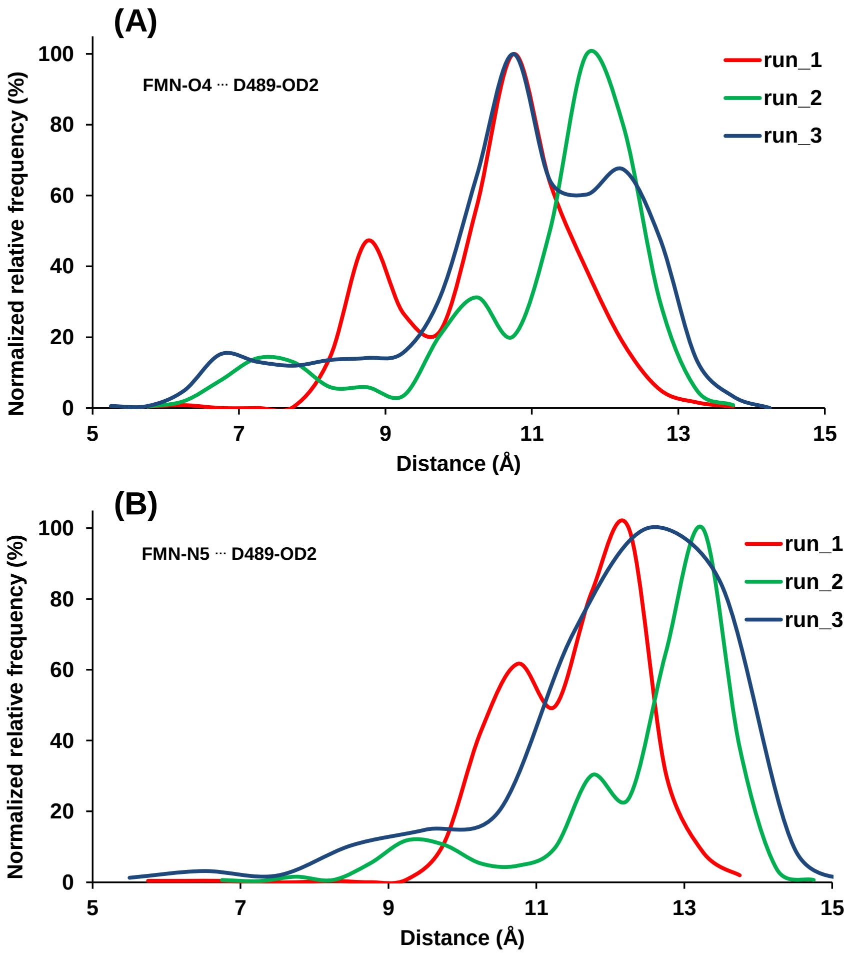

Figure S19: Distance distribution curve of the interatomic distances for the indicated residue (atom) pairs (in Å) calculated over the MD trajectories of iLOV-Q489D variant with deprotonated D489; (A) FMN-N5 … D489-OD2 distance distribution, (B) FMN-O4 … D489-OD2 distance distribution for three independent 50 ns MD runs (run_1, red; run_2, green; run_3, blue). The data was used to generate the distance distribution curves shown in Figure S8, C (light grey and dark grey lines).

Supporting References

1. Cao Z, Buttani V, Losi A, & Gärtner W (2008) A blue light inducible two-component signal transduction system in the plant pathogen Pseudomonas syringae pv. tomato. *Biophysical journal* 94(3):897-905.
2. Davari MD*, et al.* (2016) Photophysics of the LOV-Based Fluorescent Protein Variant iLOV-Q489K Determined by Simulation and Experiment. *The journal of physical chemistry. B* 120(13):3344-3352.
3. Christie JM*, et al.* (2012) Structural tuning of the fluorescent protein iLOV for improved photostability. *Journal of Biological Chemistry* 287(26):22295-22304.
4. Krieger E, Koraimann G, & Vriend G (2002) Increasing the precision of comparative models with YASARA NOVA—a self-parameterizing force field. *Proteins: Structure, Function, and Bioinformatics* 47(3):393-402.
5. Van Durme J*, et al.* (2011) A graphical interface for the FoldX forcefield. *Bioinformatics* 27(12):1711-1712.
6. Guerois R, Nielsen JE, & Serrano L (2002) Predicting changes in the stability of proteins and protein complexes: a study of more than 1000 mutations. *Journal of molecular biology* 320(2):369-387.
7. Case D*, et al.* (2015) AMBER 2015. *University of California, San Francisco*.
8. Hornak V*, et al.* (2006) Comparison of multiple Amber force fields and development of improved protein backbone parameters. *Proteins: Structure, Function, and Bioinformatics* 65(3):712-725.
9. Cornell WD*, et al.* (1995) A second generation force field for the simulation of proteins, nucleic acids, and organic molecules. *Journal of the American Chemical Society* 117(19):5179-5197.
10. Wang J, Wolf RM, Caldwell JW, Kollman PA, & Case DA (2004) Development and testing of a general amber force field. *Journal of computational chemistry* 25(9):1157-1174.
11. Olsson MH, Søndergaard CR, Rostkowski M, & Jensen JH (2011) PROPKA3: consistent treatment of internal and surface residues in empirical p K a predictions. *Journal of Chemical Theory and Computation* 7(2):525-537.
12. Jorgensen WL, Chandrasekhar J, Madura JD, Impey RW, & Klein ML (1983) Comparison of simple potential functions for simulating liquid water. *The Journal of chemical physics* 79(2):926-935.
13. Essmann U*, et al.* (1995) A smooth particle mesh Ewald method. *The Journal of chemical physics* 103(19):8577-8593.
14. Humphrey W, Dalke A, & Schulten K (1996) VMD: visual molecular dynamics. *Journal of molecular graphics* 14(1):33-38.
15. Schrodinger L (2010) The PyMOL molecular graphics system, version 1.3 r1.
16. Hess B, Kutzner C, Van Der Spoel D, & Lindahl E (2008) GROMACS 4: algorithms for highly efficient, load-balanced, and scalable molecular simulation. *Journal of Chemical Theory and Computation* 4(3):435-447.
17. Holzer W*, et al.* (2005) Photo-induced degradation of some flavins in aqueous solution. *Chem Phys* 308(1-2):69-78.
18. Kowalczyk RM, Schleicher E, Bittl R, & Weber S (2004) The photoinduced triplet of flavins and its protonation states. *J Am Chem Soc* 126(36):11393-11399.
19. Stoll S & Schweiger A (2006) EasySpin, a comprehensive software package for spectral simulation and analysis in EPR. *J Magn Reson* 178(1):42-55.
20. Stoll S & Schweiger A (2007) Easyspin: Simulating CW ESR spectra. *ESR Spectroscopy in Membrane Biophysics,* BIOLOGICAL MAGNETIC RESONANCE, eds Hemminga MA & Berliner LJ (Springer, New York), Vol 27, pp 299-321.
21. Rodgers CT & Hore PJ (2009) Chemical magnetoreception in birds: the radical pair mechanism. *Proceedings of the National Academy of Sciences of the United States of America* 106(2):353-360.
